# Effect of shared information and owner behavior on showing in dogs (*Canis familiaris*)

**DOI:** 10.1007/s10071-020-01409-9

**Published:** 2020-07-05

**Authors:** Melanie Henschel, James Winters, Thomas F. Müller, Juliane Bräuer

**Affiliations:** 1 Department for General Psychology and Cognitive Neuroscience, Friedrich Schiller University Jena, Jena, Germany; 2 Department of Linguistic and Cultural Evolution, Max Planck Institute for the Science of Human History, 10, Kahlaische Straße, 07745 Jena, Germany; 3 Minds and Traditions Research Group, Max Planck Institute for the Science of Human History, 10, Kahlaische Straße, 07745 Jena, Germany

**Keywords:** Dog–human interaction, Dogs, Communication, Showing, Shared information

## Abstract

Dogs’ production of referential communicative signals, i.e., *showing*, has gained increasing scientific interest over the last years. In this paper, we investigate whether shared information about the present and the past affects success and form of dog–human interactions. Second, in the context of showing, owners have always been treated as passive receivers of the dog’s signals. Therefore, we examined whether the owner’s behavior can influence the success and form of their dog’s showing behavior. To address these questions, we employed a hidden-object task with knowledgeable dogs and naïve owners. Shared information about the present was varied via the spatial set-up, i.e., position of hiding places, within dog–owner pairs, with two conditions requiring either high or low precision in indicating the target location. Order of conditions varied between pairs, representing differences in shared knowledge about the past (communication history). Results do not support an effect of communication history on either success or showing effort. In contrast, the spatial set-up was found to affect success and choice of showing strategies. However, dogs did not adjust their showing effort according to different spatial set-ups. Our results suggest that the latter could be due to the owner’s influence. Owner behavior generally increased the effort of their dog’s showing behavior which was stronger in the set-up requiring low showing precision. Moreover, our results suggest that owners could influence their dog’s showing accuracy (and thereby success) which, however, tended to be obstructive.

**Electronic supplementary material:**

The online version of this article (10.1007/s10071-020-01409-9) contains supplementary material, which is available to authorized users.

## Introduction

Communication, most simply defined, is the transfer of information from one entity to another, most commonly described in terms of the sender–receiver model (Shannon and Weaver 1949). A mutual understanding about signs and rules of how to use them is essential to achieve success in this process (e.g., Stevens 1950). Based on this, developing successful communication across species borders can be viewed as a particularly difficult endeavor, since the agreement upon signs and rules has to be brought in line with already existing but potentially very different communication systems between these two species to avoid misunderstandings (e.g., the common misinterpretation of the chimpanzee fear grin as happy smile; e.g., Aldrich 2015; 2018; Keeley 2004). A number of theoretical accounts describe communication systems that allow for new signals to develop between species, including ontogenetic ritualization (e.g., Tomasello and Call 1997; Tomasello et al. 1994), co-construction of meaning (e.g., Bard et al. 2019) and invented signals (e.g., Fröhlich and van Schaik 2020), but arguably doing so is considerably more difficult than intraspecific communication. Even so, we can observe one powerful example of interspecies communication almost daily: communication between dogs and humans.

The essence of the *domestication hypothesis* is that, through the evolutionary pressures of their long coexistence, dogs have become particularly skilful in communicating with humans. This is supported by a growing body of research (Hare et al. 2002; Marshall-Pescini and Kaminski 2014; Miklósi et al. 2003; Piotti and Kaminski 2016; Topál et al. 1998; but see also Udell et al. 2010) and includes dogs’ outstanding sensitivity to communicative behaviors on the part of humans, such as pointing or eye gaze (Hare et al. 2002; Hare and Tomasello 2005; Kaminski et al. 2012; Miklósi and Soproni 2006). Dogs’ sensitivity to human-given cues is so pronounced that they sometimes respond even when there is no communicative intent from the human. For instance, Lit et al. (2011) demonstrated that scent detection dogs are more likely deceived by their human handlers’ beliefs about scent locations than decoy smells (food and toys). Although Udell et al. (2010) outline valid points supporting ontogenetic learning, especially during sensitive developmental phases, as source of this heightened sensitivity of dogs towards human communicative cues, currently more experimental evidence exists in favor of the domestication hypothesis than against it. Therefore, we chose this hypothesis as the basis of our current study. Nonetheless, it is important to emphasize that this does not exclude the principal possibility of ontogenetic learning playing a role in human–dog communication (see also Sect. “Discussion”).

### Showing

But dogs are also able to successfully send out communicative signals towards humans themselves. One specific behavior, which has received considerable attention, is known as showing (Gaunet 2008, 2010; Gaunet and Deputte 2011; Gaunet and El Massioui 2014; Heberlein et al. 2016, 2017; Kaminski et al. 2011; Marshall-Pescini et al. 2009; Miklósi et al. 2005; Passalacqua et al. 2011; Piotti and Kaminski 2016; Savalli et al. 2014; Virányi et al. 2006). Miklósi et al. (2000) were the first to specifically investigate showing behavior in dogs. They defined showing as “a communicative action consisting of both a *directional component* related to an external target and an *attention-getting component*, that directs the attention of the perceiver to the informer or sender” (Miklósi et al. 2000, p. 159, emphases added). Miklósi et al. let dogs witness how a piece of food or a toy was hidden out of their reach. Afterwards, their naïve owner entered the room, instructed to find the hidden object with the help of their dog. To control for mere motivational or audience effects, two other conditions were implemented in which either only dog and hidden object or only dog and owner were in the room. In all conditions, the authors coded the occurrence of a number of dog behaviors, most importantly vocalizations and gazing at the hiding place and the owner, which were also subjected to a sequential analysis (*gaze alternation*). Miklósi et al. could show that, without previous training, dogs used gaze alternation as well as vocalizations, seemingly to signal the location of the hidden object to their owners.

The interest in showing behavior is mainly based on the suggestion that for species without hands, gaze alternation, i.e., repeated moving of gaze direction between target and receiver (Gómez 1990), which often accompanies pointing in humans (e.g., Bruinsma et al. 2004), could be functionally analogous to pointing and, therefore, intentional and referential (Harding and Golinkoff 1979; Leavens and Hopkins 1998; Leavens et al. 2005). In Miklósi et al.’s (2000) study, showing behavior only occurred in the presence of both owner and food or toy, indicating that showing behavior in dogs is indeed a form of functionally referential communication. Later studies confirmed that showing behavior in dogs fulfills all criteria (Leavens 2004; Leavens et al. 2005) of intentional referential communication (Gaunet 2010; Gaunet and Deputte 2011; Heberlein et al. 2017; Savalli et al. 2014; Virányi et al. 2006).

In contrast to gaze alternation, vocalizing in itself does not qualify as showing, but rather represents an attention-getting component. Barking and whining have been described as attention-capturing signals in the past (Bekoff 1974; Bradshaw and Nott 1995; Fox 1971). In addition to gazing and vocalizing, other components of showing exhibited by dogs in hidden-object tasks have been identified in Miklósi et al.’s (2000) and other studies. Directional components include moving towards (Heberlein et al. 2016, 2017) or spending time near the hiding place, i.e., using their own position as a local enhancement cue (Gaunet and Deputte 2011; Hare et al. 1998; Miklósi et al. 2005; Savalli et al. 2014), manipulating (Gaunet 2010; Miklósi et al. 2005; Savalli et al. 2014), sniffing (Gaunet 2010; Miklósi et al. 2000; Savalli et al. 2014) or jumping at the hidden object (Hare et al. 1998); and as attention-getting components include establishing body contact with the owner (Gaunet 2008, 2010; Gaunet and Deputte 2011; Heberlein et al. 2016, 2017; Savalli et al. 2014) and moving towards the owner (Heberlein et al. 2016, 2017).

It is important to emphasize that in all studies investigating showing, owners could overall successfully use the signals provided by their dogs to find the hidden objects. Thus, showing behavior provides a powerful example of successful dog–human communication. However, in all studies on this topic so far, owners have been treated as passive receivers of the dog’s showing signals. Therefore, an interesting question remains unanswered: whether owners can influence showing behavior, and thereby maybe even success as well. The above-mentioned studies outlining dog’s sensitivity to human communicative signals suggest an influential potential for owners here as well.

### Shared information and the principle of least effort

Past studies with human participants have shown that shared information between communication partners influences the form their communication takes and its success, for instance when both communication partners remember past discourse or share access to information in the present such as stimuli attributes (Brennan and Clark 1996; Brown-Schmidt et al. 2008, 2009; Krauss and Weinheimer 1967; Müller et al. 2019; Winters et al. 2015, 2018; Yoon et al. 2016). Apart from its positive influence on communication success, relying on shared information also often allows interlocutors to reduce their communicative effort. Zipf (1949) argued that human behavior in general is guided by the principle of least effort, that is, people try to spend as little effort as possible on the problems they face by taking current and future situations into account. This strategy proved to be effective on the individual as well as the collaborative level (Clark and Wilkes-Gibbs 1986).

Taken together, this implies that humans use shared information to optimize effort and, thus, achieve an optimal trade-off between efficiency and communicative success. At this point, there is relatively little empirical work demonstrating whether these factors influence communication in species other than humans or even cross-specific communication.

In a study by Scheider et al. (2011), dogs searched longer and more often at an empty location a human pointed at, in a condition in which they had previously found food in the presence of that human, than dogs without such context information. This study demonstrates that such additional information not only affects dogs’ behavior but also their interpretation of human communication. However, this study investigated searching/choice behavior and not showing. Heberlein et al. (2017) delivered an indication that showing might be sensitive to shared information between dog and human as well. They found that dogs exhibited less showing behavior if the human partner and the dog shared the knowledge about the correct hiding location, in contrast to when only the dog observed the hiding procedure. Furthermore, in a study by Gaunet and Deputte (2011), dogs positioned their bodies differently depending on the height of the target location. This study is particularly relevant since shared information represents the spatial layout of the experimental set-up, like in the current study. However, the findings of Gaunet and Deputte (2011) only illuminate the sensitivity of a (directional) component of showing behavior to the spatial set-up. Thus, it remains unclear whether and to what degree showing behavior as a whole is affected by the spatial set-up.

At the time of this study, no research could be found that specifically investigated whether dogs follow the principle of least effort in their communication in general or with humans in particular. However, generally, behavior research in humans as well as non-human animals has adopted the idea that organisms strive to save energy and minimize effort (Menzel 1973; Mowrer and Jones 1943; Sparrow and Newell 1998; Tsai 1932; Waters 1937). Moreover, several studies found Zipf’s (1949) principle of least effort to apply to animal communication (Doyle et al. 2011; Hanser et al. 2004), specifically in dolphins (Ferrer-i-Cancho and Lusseau 2009; McCowan et al. 1999), squirrel monkeys (McCowan et al. 2002), formosan maquaques (Semple et al. 2010), bats (Luo et al. 2013), and to some extent common marmosets (Ferrer-i-Cancho and Hernández-Fernández 2013) which speaks in favor of the generalizability of Zipf’s principle of least effort.

In general, the long commensal history of dogs and humans is suggested to have driven dogs to develop communication patterns that follow the same rules as those of humans (Fitch et al. 2010; Miklósi et al. 2004; Schleidt and Shalter 2003; Topál et al. 1998). Accordingly, crucial factors that have been found for human communication could apply to dog–human communication as well.

### The present study

In the current study, we examined whether present and past shared information between dogs and their owners, as well as the owner’s behavior, influence the form and the success of human–dog interactions in a hidden-object task. The set-up, similar to Miklósi et al. (2000), enabled only dogs to witness the hiding of their toy while owners re-entered the room afterwards. Thus, dogs had to show their owners where the toy had been hidden to get it back and play with their owners. Two different conditions manipulated present shared information in the form of the spatial set-up: The distance between the possible hiding places was either small (*close* condition) or big (*far* condition), therefore requiring either high or low precision in indicating of the target location. The order in which pairs went through these conditions represented different communication histories, i.e., shared information about the past. Dogs could make use of information about the present (i.e., condition) as well as their memories from their first session (i.e., communication history) to adjust their communication strategies which in turn might influence success in finding the hidden toy.

### Hypotheses

Building on the aforementioned literature, four hypotheses are proposed regarding the communicative behavior between dogs and their owners:

#### H1: success of communication

Dogs are able to successfully show the location of the hidden object to their owner. Based on this hypothesis, we predict that a greater proportion of showing referring to the correct location predicts greater success.

#### H2: spatial set-up

The distance between the boxes affects success and form of dog–owner communication. Regarding this hypothesis, we predict that (a) performance will be better in the far compared to the close condition, i.e., there will be a main effect of condition, and that (b) the form of communication will differ between conditions.

#### H3: communication history

Past interactions between dogs and owners constrain future communicative behaviors. Based on this hypothesis, we predict that (a) the starting condition determines the showing strategy dogs use throughout the whole procedure, i.e., dogs use relatively more high-effort strategies starting with close than with far. This has direct implications concerning performance: (b) pairs perform better if they start with close than when they start with far. Thus, we expect an interaction between condition and session regarding showing effort as well as performance (an in-depth description of these predictions can be found in Online Resource 1).

#### H4: principle of least effort

Dogs always use the minimal effort strategy for a given context. Here, the prediction is that the far condition should be characterized by relatively less high-effort strategies than the close condition. This should hold irrespective of the order in which pairs completed the conditions. Thus, this hypothesis predicts a main effect of condition regarding showing effort, but no interaction of condition and session.

Note that H3 and H4 contradict each other. Although the influence of communication history and the principle of least effort are not necessarily mutually exclusive, in this set-up we wanted to examine the isolated contributions of the two factors.

In addition to these hypotheses, the set-up of the study also gave the opportunity to look at the interaction of dogs’ and owners’ behaviors. Regarding this part of the showing paradigm, however, existing literature does not allow precise predictions. Therefore, we analyzed this relationship exploratively to provide a first look at the interactive part of showing. First, we examined whether the owners’ behavior can influence their dogs’ proportion of correct showing and thereby, indirectly, the pair’s success. Second, we examined the owner’s influence on showing effort.

## Materials and methods

### Subjects

The 32 pairs that took part in this study were normal pet dogs of various breeds and their owners. Two pairs had to be excluded during testing because of health problems of the dog, leading to a final sample size of 30 dog–owner pairs. Of these dogs, 18 were female and 12 were male (mean age 5.8 years, range 2–13 years), whereas 24 of the owners were female and 6 were male (for detailed information about pairs see Online Resource 1 and 2). Dogs were recruited from the DogStudies database of the Max Planck Institute for the Science of Human History in Jena. Selection criteria for dogs were high toy motivation and the ability to fetch inert objects (which was additionally tested explicitly; see Sect. Pretest). All dogs were healthy individuals with no known sight or hearing problems and no known aggression towards humans.

### Materials and set-up

**Fig. 1:**
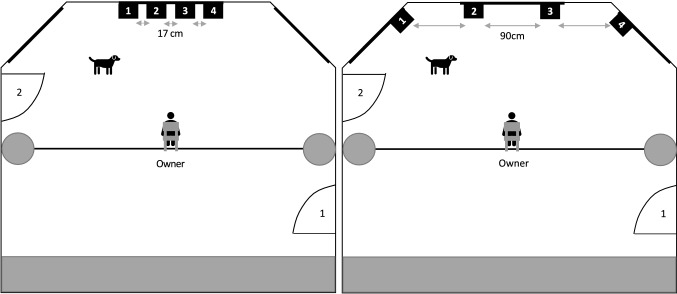
Set-up of the close condition (left) and the far condition (right). The four possible hiding places (numbered 1–4) were fixed on the windowsills of the room. The owner, seated on a chair, was positioned in the middle of the room which could be entered through two doors (1 and 2). One camera was positioned above the boxes, one was on the sideboard behind the owner

In the test room, four small boxes (8 cm × 15 cm × 20 cm) were attached to the windowsills which constituted the four possible hiding places. They were numbered from 1 to 4, so the owners could identify each box for their choices in the test. In the close condition, boxes were put up 17 cm apart from each other, while in the far condition, boxes were positioned 90 cm apart from each other (Fig. 1). For the owners, a chair with an accompanying questionnaire was placed in the middle of the room on which owners had to check the supposed target box (for detailed information about materials and set-up see Online Resource 1).

### Procedure

Each dog–owner pair visited the laboratory twice within 1 week. Only one condition, comprising four trials, was tested per session (i.e., per day) with an inter-trial break of ~10 min. Conducting all eight trials in one session was decided to be too demanding for most dogs. While owners were instructed for the test, dogs could freely explore the test room.

#### Pretest

Before the actual test, a pretest was conducted. The owner was instructed to sit on the chair facing the dog sitting in ~2 m distance. If necessary, one experimenter held the dog by their collar. The dog’s favorite toy was now put between the two parties and the owner was instructed to call the dog to bring the toy. Owners were told to do so in a natural manner, as they would in a typical playing or training context since the aim of this study was to investigate the typical communication of the pairs. If the dog did as requested, the pair was allowed to play for a short amount of time. The exact duration of play varied between subjects because of different play styles but was kept approximately constant within subjects to avoid unintended differential rewarding (e.g., we either kept constant how often the toy was thrown and fetched or, if pairs preferred other play styles like tug-of-war, the duration of play time was kept constant). If a dog failed to bring the toy right away it had one more chance to do the task correctly before being excluded from the study. All dogs successfully completed the pretest. This procedure was repeated at the beginning of each new trial to re-establish the play context.

#### Test

Immediately after the short play session, the owner handed over the toy to experimenter 1 (E1). Experimenter 2 (E2) left the room with the owner through door 1 (see Fig. 1).^1^ Now, E1 first gently waved the toy in front of the dog’s face to get its attention (this was repeated whenever the dog averted its gaze from the toy, accompanied by calling the dog by its name). E1 then put the toy into the target box and closed it. Immediately, the box was reopened and this procedure was repeated one more time to assure that the dog really processed where the toy had been hidden. Meanwhile, E2 guided the owner around the room to door 2 (see Fig. 1) and waited for the signal from E1 which was given as soon as E1 had closed the box and left the room. E2 now opened the door and let the owner inside the room.

We also wanted to investigate the effect of behavioral restrictions on communication. Previous research has shown that a standardized, but nevertheless unnatural setting, can inhibit dogs’ natural behavior and conceal their actual abilities (e.g., Bräuer et al. 2013). Therefore, the following procedure was divided into two phases with differing degrees of standardization (since this manipulation hardly yielded any effects, most results will not be discussed here and can be viewed in Online Resource 1).

Phase 1: The owner entered the room and directly sat down on the chair. During phase 1, owners were not allowed to stand up and walk around. Other than that, no constraints were put on communication between dogs and owners. After 1 min had elapsed, one of the experimenters signaled the owner from outside the room to fill in the questionnaire which also marked the end of phase 1. The owner now had to check the box in which he or she assumed the toy is located (i.e., make their choice for phase 1).

Phase 2: As soon as the owners had checked the questionnaire, they were allowed to stand up and move around freely within the test room. Here, the only communicational constraint was that owners were not allowed to open the boxes unless they wanted to make a choice. Phase 2 lasted a maximum of 1 min, hence, in contrast to phase 1, owners had the possibility to make their choice before 1 min had passed, even directly after filling in the questionnaire without further interacting with their dog. However, if owners had not opened a box after 1 min, experimenters prompted them by calling “Wählen!” (German for “Choose!”). The box that was opened in phase 2 could be different from the choice made in phase 1. If the pair chose correctly in phase 2, they could play together as a reward (again duration of play varied across but not within subjects). If the wrong box was opened, the experimenters would enter the room and open the correct box to show the toy to both the dog and the owner, but the pair was not allowed to play.^2^ Choices for phase 2 were coded live by the experimenters and back-checked from tape afterwards.

In contrast to previous studies, the current set-up only prevented smaller dogs from accessing the boxes. Consequently, some dogs retrieved the toy on their own.^3^ If dogs retrieved the toy already in phase 1, before owners could check the questionnaire, the respective box was taken as choice for both phases because in this case it was unambiguous for the owner which box was the correct one. If owners did not check a box on the questionnaire and the dog did not retrieve the toy, the choice for phase 1 was coded as *0* and subsequently as incorrect choice because it neither overlapped with the target box nor did it indicate a correct inference from the dog’s behavior. In between trials dogs had no access to their favorite toy or any other toys. Online Resource 3 displays a video of the procedure.

### Design

Order of conditions was counterbalanced across subjects. Order of boxes was semi-randomized across conditions, with the stipulations that the same box could not be target in two consecutive trials within a session and that each box had to be target twice for each dog. The number of the first box was counterbalanced across subjects. Due to excluded pairs and problems during the test, the final distribution is slightly uneven: Seven pairs started with box 1, ten with box 2, six with box 3 and seven with box 4.^4^

### Behavioral coding

All behaviors were coded using Solomon Coder software (Péter 2017) which was set-up with a sensitivity of 0.20 s. For dogs, seven different behaviors were coded: gazes directed at each of the boxes and the owner, movements directed at each of the boxes and the owner, time spent near each box, jumping/standing upright in front of each of the boxes, vocalizations, whether the dog opened the boxes and whether the dog retrieved the toy on its own.

For owners, the following behaviors were coded into one variable *owner behavior*: owners’ gazing at the dog, gazing at the boxes (i.e., one specific box or the general direction of the boxes), pointing at the boxes, nodding in the direction of the boxes, showing empty hands, shrugging, approaching the boxes, talking (any utterances by the owner, i.e., including laughing, sneezing, coughing) and calling the dog by its name (including obvious nickname versions of the dog’s name, e.g., Sue for Susi, but no other kinds of nicknames that were given, e.g., honey). This variable is very broadly defined, since, for an explorative analysis of the interaction of owner and dog, the variable should cover a wide range of possibly influential behaviors. (We also conducted analyses with owner behavior separated into non-verbal prompting, talking and calling the dog’s name which can be seen in Online Resource 1).

All dog- and owner-related variables were coded in terms of frequency, and time point relative to all other behaviors (both the dog’s and the owner’s), i.e., how often and when they happened. All behaviors that were necessary for the calculation of showings (see below) were additionally coded in terms of duration, i.e., when they started and when they ended relatively to all other behaviors. Solomon Coder provides a timetable of all behaviors (dog’s and owner’s) as output as well as automatically calculates frequencies and durations of variables.

To assess the inter-coder reliability, 20% of the videos (i.e., 6 pairs) were coded by a second observer, naïve to the hiding location and the purpose of the study. Agreement between the two coders was calculated using Spearman rank order correlation, and inter-coder reliability was assessed according to the limits proposed by Cicchetti (1994). Accordingly, mean inter-observer reliability was good for frequencies of gazes (*r* = 0.74), and excellent for durations of gazes (*r* = 0.82), frequencies (*r* = 0.78) and durations of the dog’s movements (*r* = 0.82), frequencies (*r* = 0.97) and durations (*r* = 0.96) of dogs spending time near each box, frequencies (*r* = 0.98) and durations (*r* = 0.99) of jumping/standing upright at the boxes as well as opening boxes (*r* = 0.92) (Spearman rank order correlation coefficients for each behavior per box can be seen in Online Resource 1). For dog vocalizations, coders reached good agreement for frequencies (*r*_s_ = 0.74, *p* < 0.001) and excellent agreement for durations (*r*_s_ = 0.77, *p* < 0.001). Lastly, inter-coder reliability was excellent for owner behavior (*r*_s_ = 0.97, *p* < 0.001).

To specify showing behaviors, we generalized the definition for gaze alternation that Russell et al. (1997) initially used for chimpanzees and Miklósi et al. (2000) transferred to dogs, to include other showing behaviors as well: The directional component has to be followed directly and within two seconds by the attention-getting component or vice versa (i.e., order of components does not matter). Therefore, the above-mentioned behaviors were divided into directional components and attention-getting components (Miklósi et al. 2000). All 15 possible combinations of these components form the showing behaviors analysed in this study (see Table 1).

**Table 1:** List of combinations of directional components and attention-getting components forming showing.

Directional component	Attention-getting component
Gaze at box	Gaze at owner
	Move towards owner
	Vocalize
Move towards box	Gaze at owner
	Move towards owner
	Vocalize
Time near box	Gaze at owner
	Move towards owner
	Vocalize
Jump/stand upright in front of box	Gaze at owner
	Move towards owner
	Vocalize
Open box	Gaze at owner
	Move towards owner
	Vocalize

Both alternations and overlaps of directional and attention-getting components classify as showing. For alternations, order of the two components does not matter.

Since the initial definition focused on gaze alternation (Miklósi et al. 2000; Russell et al. 1997), it stated that the two components have to occur in succession. In this study, however, the two components could also occur simultaneously (e.g., spending time near a box while gazing at the owner). Therefore, both alternations and (partial or complete) overlaps of the above-mentioned behaviors were defined as showing. Showings were calculated based on the timetables provided by Solomon Coder using a script programmed with Python (further details regarding behavioral coding, flowcharts. depicting the employed algorithm and an example of the generated output can be seen in Online Resource 1).

For analyses regarding showing effort, low-effort showing was defined as the least effortful showing strategy: gazing at a box plus gazing at the owner (i.e., gaze alternation). Similarly, high-effort showings were defined as all showings involving the most effortful behavioral component: jumping/standing upright plus any of the three attention-getting components (i.e., gazing at the owner, moving towards the owner or vocalizing). However, since many dogs did not exhibit jumping/standing upright at all, the second most effortful showing strategy was added as well: moving towards a box plus moving towards the owner.

### Statistical analysis

All analyses were done with R software (version 3.6.3; R Core Team 2020), the code can be viewed in Online Resource 4. In line with the Cumming’s propositions of “new statistics” (Cumming 2014, p. 7) and the Publication Manual of the American Psychological Association (APA 2010), raw estimations and effect sizes will be reported and discussed independent of, and in addition to, their significance status (*α* = 0.05) and with regard to their respective confidence intervals. Raw data can be found in Online Resource 2. Results of analyses adjusted for outliers are displayed in Online Resource 1.

Overall success, i.e., whether pairs chose correctly or not, was investigated with a one-sample *t* tests against chance (25%) for each phase since two different measures of performance were used in phase 1 and 2 (i.e., questionnaire versus opening box). Two-sided, paired *t* tests were calculated to assess differences in performance between phases and differences in frequencies of the different showing types between conditions.

For all other effects, we applied a model comparison approach. Models were compared based on their respective Akaike information criterion (AIC; Akaike 1974) value. The respective model with the smallest AIC was chosen as final model, and to test for significant differences between the models a Chi-square test was applied (results of all calculated models and comparisons can be found in Online Resource 1). Whenever the program responded a warning of nonconvergence, the respective model was optimized using the BOBYQA algorithm (Powell 2009). For each analysis, rows including missing values for a variable of interest were excluded. According to the study design, session, trial and phase were always treated as one nested variable (i.e., phases were nested within trials which were nested within sessions) which is henceforth referred to as *time*.

To investigate the effects on success, generalized linear mixed-effects models (GLMM) with a binomial distribution were calculated using the R package lme4 (version 1.1–19; Bates et al. 2018). Since the outcome variable was binary (i.e., correct vs. incorrect), a logit transformation was applied, i.e., the dependent variable for models was the probability of pairs choosing correctly rather than incorrectly.

For the investigation of effects on the proportion of correct showing and showing effort, linear mixed-effects models (LMM) were calculated. For this, we used the R package lme4 (version 1.1–19; Bates et al., 2018), and *p *values were calculated using the lmerTest package (version 3.0–1; Kuznetsova et al. 2017). Showing effort was defined as the frequency of high-effort showings relative to the sum of frequencies of high- and low-effort showings, i.e., the proportion of high-effort showing. Hence, higher values for this variable indicate higher showing effort.

## Results

### Overall success

Results show that pairs as a group chose correctly significantly above chance level in phase 1 (*M* = 53.75, SD = 24.82, *t*[29]= 11.81, *p* < 0.001, Cohen’s *d* = 2.16, 95% CI [1.23, 3.05]) as well as in phase 2 (*M* = 59.58, SD = 24.93, *t*[29] = 13.04, *p* < 0.001, Cohen’s *d* = 2.38, 95% CI [1.42, 3.31]). Moreover, both of these effects are of substantial size. Performance in phase 2 was significantly better than in phase 1 (*t*[29]  = −2.25, *p* = 0.032, Cohen’s *d* = 0.23, 95% CI [−0.28, 0.74]).

### Distribution of showing types

Overall, showings involving gazing, moving or spending time near a box were used more readily than showings involving vocalizing, jumping/standing upright or opening boxes (see Table 2). But interestingly, those behaviors that were used less often by dogs corresponded more with the target box and the owner’s choice than behaviors dogs exhibited more frequently (see Table 2).

**Table 2:** Mean frequency, accuracy and choice rate of each type of showing behavior of a pair per phase within a trial

Directional component	Gazing at owner	Moving towards owner	Vocalizing
Frequency			
Gazing at a box	9.29	0.99	0.46
Moving towards a box	3.42	4.10	0.36
Spending time near a box	**12.38**	7.52	0.83
Jumping/standing upright at a box	0.57	0.37	0.11
Opening a box	–^a^	0.03	*0.01*
Accuracy			
Gazing at a box	0.38	0.36	0.43
Moving towards a box	0.30	0.31	0.47
Spending time near a box	0.29	*0.25*	0.31
Jumping/standing upright at a box	0.53	0.53	0.68
Opening a box	–^a^	0.58	**1.00**
Choice rate			
Gazing at a box	0.39	0.33	0.42
Moving towards a box	0.33	0.33	0.53
Spending time near a box	0.30	*0.26*	0.35
Jumping/standing upright at a box	0.64	0.65	0.77
Opening a box	–^a^	0.67	**1.00**

The highest numbers are written in bold, the lowest in italics

^a^Opening a box plus gazing at the owner was not exhibited at all

Dogs used the following showing strategies significantly more often in the far condition: gaze alternation (i.e., gazing at box plus gazing at owner; far: *M* = 9.84, SD = 7.34, close: *M* = 8.75, SD = 6.96, *t*[239] = 1.99, *p* = 0.048, Cohen’s *d* = 0.15, 95% CI [−0.03, 0.33]), moving towards box plus gazing at owner (far: *M* = 3.68, SD = 3.25, close: *M* = 3.16, SD = 3.25, *t*[239] = −2.33, *p* = 0.021, Cohen’s *d* = 0.16, 95% CI [−0.02, 0.34]) and moving towards box plus moving towards owner (far: *M* = 4.69, SD = 4.57, close: *M* = 3.51, SD = 4.01, *t*[239] = −4.64, *p* < 0.001, Cohen’s *d* = 0.27, 95% CI [0.09, 0.45]). Strategies that were exhibited significantly more often in the close condition were: spending time near box plus gazing at owner (close: *M* = 12.97, SD = 8.62, far: *M* = 11.79, SD = 7.43, *t*[239] = 2.58, *p* = 0.010, Cohen’s *d* = 0.15, 95% CI [−0.03, 0.33]) and spending time near the box plus vocalizing (close: *M* = 1.04, SD = 3.18, far: *M* = 0.62, SD = 1.73, *t*[239] = 2.30, *p* = 0.022, Cohen’s *d* = 0.16, 95% CI [−0.01, 0.34]). No significant differences were found for the other showing types (see Online Resource 1), indicating similar distributions of these strategies in the two conditions.

### Effect of correct showing, condition and time on success

The final model describing the effect of the proportion of correct showing on success (prediction 1b) displayed a large and significant effect of correct showing (*β* = 6.81, SE = 1.03, *z* = 6.62, *p* < 0.001). Thus, a higher proportion of correct showing significantly increased the probability of choosing the correct box. Additionally, time showed a significant effect of trial in session 1 (*β* = −0.40, SE = 0.15, *z* = −2.64, *p* = 0.008) indicating a decline in performance over trials in session 1.

**Fig. 2:**
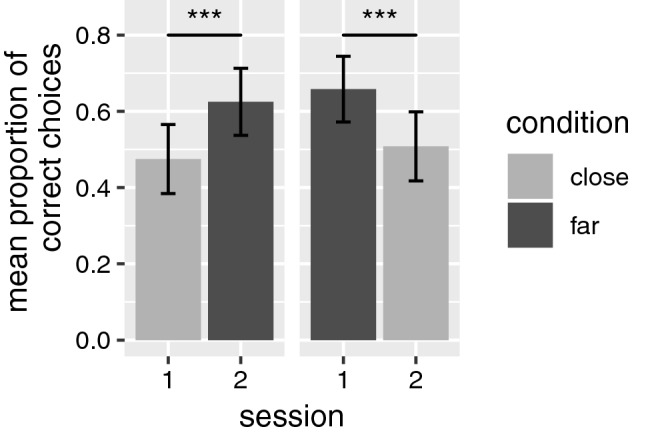
Mean proportion of correct choices (with standard errors) by condition and session. Note that the differences across sessions within a condition (e.g., close in session 1 vs. close in session 2) did not reach significance, neither for close, nor for far. Significance code: ***p < 0.001

Results of the final model investigating the effect of condition (prediction 3), time and their interaction on success (prediction 4b) show a significant main effect of condition (*β* = 0.78, SE = 0.21, *z* = 3.71, *p* < 0.001), i.e., pairs performed better in the far condition than in the close condition (see Fig. 2). Moreover, time showed a significant effect of trial in session 1 again (*β* = −0.47, SE = 0.14, *z* = −3.21, *p* = 0.001), indicating that there was a significant decline in the performance over trials in session 1. Conversely, since adding the interaction of condition and time did not improve the model, this suggests that the performance in a respective condition did not depend on whether it was completed first or second (see Fig. 2).

### Effect of condition and time on showing effort

Results of the final model for the effect of condition (prediction 5), time and their interaction (prediction 4a) on showing effort displayed a significant effect of phase in session 1 (*β* = −0.05, SE = 0.01, *t*[420.37] = −6.43, *p* < 0.001) and 2 (*β* = −0.05, SE = 0.01, *t*[420.75] = −6.65, *p* < 0.001), suggesting that, in both sessions, showing effort was significantly higher in phase 1 than in phase 2. Thus, overall, time displayed a significant effect on showing effort. In contrast, since model comparisons revealed that the addition of condition as main effect or interaction did not improve the model, the implication is that there is no effect of condition or its interaction with time on showing effort.

### Correlation between showing accuracy and seconds

Showing accuracy (i.e., whether the respective showing behavior referred to the correct box or not) correlated significantly with seconds passed within a respective trial (*r* = 0.016, *t*[19410] = 2.24, *p* = 0.025, 95% CI [0.002, 0.030]). This indicates that showing accuracy very slightly increased with passing time.

### Effect of owner behavior on correct showing

**Fig. 3:**
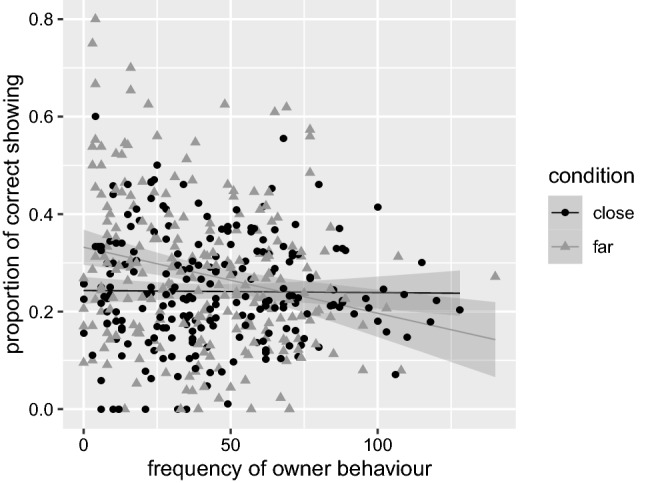
Proportion of correct showing (with standard errors) by frequency of owner behavior and condition

Owner behavior exhibited no significant main effect on the proportion of correct showing (*β* ≈ 0.00 SE ≈ 0.00, *t*[388.00] = −0.39, *p* = 0.694), indicating that the owner’s behavior did not overall benefit or worsen the dog’s proportion of correct showing. However, owner behavior significantly interacted with condition (*β* ≈ 0.00, SE ≈ 0.00, *t*[436.70] = −2.75, *p* = 0.006) in that the owner’s influence impeded the dog’s correct showing in far but hardly affected it in close (see Fig. 3). The main effect of condition reached significance as well (*β* = 0.08, SE = 0.02, *t*[432.10] = 3.80, *p* < 0.001) indicating that the proportion of correct showing was overall higher in far than in the close condition. But due to the significant interaction the main effect should not be interpreted in isolation (Zar 1999).

### Effect of owner behavior on showing effort

**Fig. 4:**
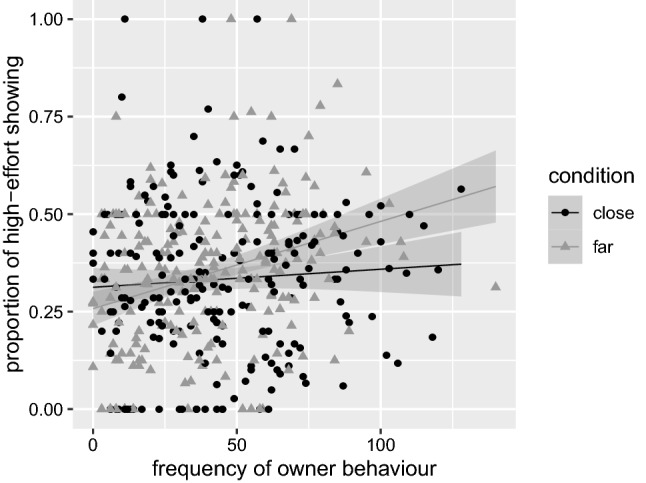
Proportion of high-effort showing (with standard errors) by frequency of owner behavior and condition

Owner behavior showed no significant main effect on showing effort (*β* ≈ 0.00, SE ≈ 0.00, *t*[384.60] = 0.19, *p* = 0.849), indicating that the owner’s behavior did not overall increase or decrease the dog’s showing effort. However, owner behavior significantly interacted with condition (*β* ≈ 0.00, SE ≈ 0.00, *t*[425.90] = 2.52, *p* = 0.012; see Fig. 4), indicating that the owner’s influence greatly increased showing effort in the far condition but hardly increased it in close. The effects of time were similar to the ones detected in the analyses concerning predictions 3a and 4.

## Discussion

The first aim of the present study was to determine the relationship of present and past shared information with strategies as well as success of dog–owner communication. Second, this study aimed at exploring the influence owners have on success and form of their dog’s showing behavior. Results demonstrated that showing behavior in dogs is a means to successfully communicate the location of a hidden object to their owner. Analyses indicated no effect of communication history, neither on showing effort nor on success. The spatial set-up affected success but not showing effort. Owner behavior was found to have an overall negative effect on correct showing and generally increased showing effort.

### Communication about the hidden object’s location

First, since we found that success rates of pairs were significantly above chance level, we could replicate the findings of previous showing studies (Gaunet 2008, 2010; Gaunet and Deputte 2011; Hare et al. 1998; Heberlein et al. 2016, 2017; Kaminski et al. 2011; Miklósi et al. 2000; Piotti and Kaminski 2016; Savalli et al. 2014; Virányi et al. 2006): dogs engage in showing behavior as defined by Miklósi et al. (2000) to successfully indicate the location of a desired hidden object to their owners. Moreover, the key to success in the current task proved to be dogs showing their owners the correct box. Results demonstrated that the more dogs addressed the target box with their showing behavior relative to all other boxes, the higher the respective pair’s chances of choosing correctly became. This effect was significant in absence of a mere improvement of performance over time. To the contrary, performance even slightly decreased over the first four trials of the experiment. Thus, the first hypothesis is supported by the current data: Dogs are able to achieve successful communication with their owners about the hiding location of their favorite toy by means of showing.

Given that the analyses demonstrated that showing behavior was the driving force in this experiment, an interesting question is whether dogs only show the correct location to their passive owners or if owners can actively influence success in this task as well. Thus, we explored whether owners had an influence on the proportion of correct showing and thereby, indirectly, on whether they found the toy or not. Results suggested that owners did indeed influence how correctly their dogs showed but not necessarily in the most obvious way. We found that owners overall negatively impacted their dog’s proportion of correct showing. In other words: the more owners pushed their dogs to show them the hidden toy, the less they showed the correct box and the more they showed just any box. This effect seemed to be stronger in far than in close. Although at this point it remains possible that the described effect rather operates in the opposite direction, i.e., the proportion of correct showing affects the owner’s behavior, this explanation seems unlikely since owners did not know the correct location in this study.

The overall detrimental effect of owners pushing their dogs fits with findings from several other studies. Range et al. (2009) found that ostensive cues (Csibra and Gergely 2009; Sperber and Wilson 1986), i.e., verbal or non-verbal signals indicating the signaller’s communicative intention, have an activating potential regarding dogs’ behavior. Some of the owners’ behaviors in the current study are ostensive cues: looking at the dog, talking in a high-pitched voice and calling the dog by its name (Topál et al. 2014). As Kaminski et al. (2011) outlined, ostensive cues like that can activate behavior, including indicative behavior (i.e., showing), diffusely. Consequently, as in the present study, Kaminski et al. observed that, if dogs do not know what exactly they are supposed to show to their owners, they show just any location. One might argue that encouragement, both verbal and non-verbal, was beneficial for most dogs in the beginning. But with time passing, ongoing asking by the owner, especially without direct reinforcement such as praise, seemed to tell the dogs that they had not displayed the right behavior yet. Thus, they did not know anymore what to show, and, therefore, tried out other options, i.e., showing other boxes. However, results of our explorative analyses did not reflect such a pattern. Since the owner’s influence did not interact with session, trial or phase, the effect of owner behaviors on dogs’ showing did not vary as a function of time. Moreover, our analyses showed that showing accuracy did not negatively correlate with time. Accordingly, a dog’s showing did not get worse over the course of a trial. Thus, the effect of owner behavior on the proportion of correct showing rather seems to be a matter of active versus passive owners as a general characteristic. In line with this, in Kaminski et al.’s study (2011), the diffuse activation effect only occurred if the hidden object was only desirable to the owner and therefore, the dog did not know what to do. It did not occur when the dog desired the hidden object. However, it is still possible that this diffuse activation effect played a role in the present study albeit not developing over time. In one example this pattern was extremely obvious because the dog responded to the owner’s cues by fetching the lids of random boxes suggesting that the dog did not know what the actual task was.

Various authors mention another factor that could explain the negative effect of the owner’s pushing on correct showing which is more or less independent of the dog’s understanding of the task: ostensive signals generally seem to induce a “ready-to-obey” attitude in dogs leading to all following signals, like pointing, being understood as a command or instruction (Kaminski et al. 2011, 2012; Kirchhofer et al. 2012; Kis et al. 2012; Topál et al. 2009; Topál et al. 2014; but see Scheider et al. 2013). Moreover, a large body of research has demonstrated that dogs have a strong tendency to abandon their own initial (usually correct) choice in favor of another option if it is ostensively cued by a human (Erdőhegyi et al. 2007; Marshall-Pescini et al. 2011, 2012; Plourde and Fiset 2013; Prato-Previde et al. 2008; Szetei et al. 2003; Topál et al. 2009). This implies that dogs either more or less blindly follow humans’ instructions or value the information provided by humans over their own knowledge. In our study, owners often pointed at boxes, asking their dogs “Is it here?”. This way, owners might have accidentally deceived their dogs into directing showing behavior at the wrong box.

In our study, however, the owner’s behavior did not simply decrease correct showing. The effect was mainly prevalent in the far condition; while in the close condition, the effect was weak or absent. A possible explanation could be the aforementioned activation effect of human ostensive signals (Erdőhegyi et al. 2007; Kaminski et al. 2011; Marshall-Pescini et al. 2011, 2012; Plourde and Fiset 2013; Prato-Previde et al. 2008; Range et al. 2009; Szetei et al. 2003; Topál et al. 2009, 2014). Dogs might have been stimulated too much by their owners for a setting as easy as the far condition. Possibly, in the close condition, a considerable amount of encouragement was necessary to motivate dogs to try to solve such a hard task, or at least not harmful. Contrarily, in the far condition, too much encouragement might have led dogs to be overly motivated and therefore exhibit diffuse (showing) behavior and/or abandon their own initial choice for the owner’s (accidentally) cued choice. Nevertheless, this interpretation of the interaction of owner behavior with condition remains highly speculative at this point and needs further investigation.

### Sensitivity to spatial set-up and communication history

The second hypothesis of this study examined sensitivity of dog–human communication to the spatial set-up of the interaction setting. The prediction that performance should be better in the far condition than in the close condition could be confirmed, supporting the hypothesized effect of the spatial set-up on success of dog–owner communication, i.e., distance between boxes did affect performance of pairs. Moreover, the form of dog–owner communication varied between conditions as well. Showing strategies that were used more often in far all contained gazing and movements as directional and attention-getting components. Conversely, showing in the close condition always contained vocalizations. However, this could also be attributed to higher excitement in the harder condition. But remarkably, in close, dogs predominantly gave their owners directions by positioning their body near the box they wanted to show. This strategy is much more precise and, therefore, adapted to the context of the close condition. In contrast, the strategies employed in far, i.e., movements and gazes, could occur from afar as well as close to the boxes and, hence, are less precise. Interestingly, showings involving jumping/standing upright or opening boxes did not vary according to the spatial set-up although owners clearly preferred them for making their choices. This marks a mismatch in communication between dogs and owners.

In summary, this study provides further evidence that, similar to the case of human interactions (Brown-Schmidt et al. 2008; Krauss and Weinheimer 1967; Müller et al. 2019; Winters et al. 2015, 2018), shared knowledge about the communicational context influences success of interspecies communication between dogs and humans as well, at least when this shared knowledge concerns spatial cues. This adds to the study by Gaunet and Deputte (2011) who delivered the first evidence that showing behavior in dogs (although their study only focused on one component of showing) might be sensitive to the spatial context of the experimental set-up.

Hypothesis 3 stated that past interactions should constrain future communicative behavior. First, results did not confirm the prediction that dogs use more high-effort strategies when they start with the close condition. Second, we predicted that performance should reflect an effect of communication history as well. However, pairs did not perform better if they started with the close condition. Thus, the results did not confirm this prediction either. Therefore, hypothesis 3 was not supported by the current data; hence, no evidence could be found that shared information about past interaction affects dog–human communication.

Based on the current findings, communication history does not seem to play the same role in dog–human communication as it does in human communication (Brennan and Clark 1996; Brown-Schmidt 2009; Yoon et al. 2016). A possible explanation could be that this hypothesis was based on the theory that, through the domestication process, dogs might have evolved a communication system analogous to that of humans (Fitch et al. 2010; Miklósi et al. 2004; Schleidt and Shalter 2003; Topál et al. 1998). However, the study by Heberlein et al. (2016) demonstrated that hand-raised and extensively socialized wolves perform just as well as dogs in a showing task. This suggests that socialization might play a bigger role in showing than domestication, i.e., the shared evolution of humans and dogs. Moreover, showing object location is commonplace in non-domesticated captive apes (e.g., Call and Tomasello 1994; Leavens and Hopkins 1998; Leavens et al. 1996; Woodruff and Premack 1979) which is also an indicator that showing behavior might be a product of ontogeny rather than phylogeny.

It is also possible that dog–owner pairs were indeed influenced by past interactions but not within the timeframe of our experiment or observable on a group level. Miklósi et al. (2000) argued in their study that dog–owner pairs might develop unique and individualized communication systems and signals, with specific reference to ontogenetic ritualization (e.g., Tomasello and Call 1997; Tomasello et al. 1994). The behavioral observations during the experiment clearly indicated individual differences in behavior, both on the part of the dog and the owner. For instance, some pairs heavily relied on vocal communication (both dog and human) whereas others almost completely relied on non-verbal communication. In addition, some owners reported having employed scenarios similar to the experimental set-up into their playing routines before and others reported encountering this kind of situation for the first time. Therefore, some pairs might already have developed individual strategies to solve such situations and others have not. Consequently, the possibility that dog–human dyads are influenced by their communication history should not be dismissed yet and should be investigated again in future studies, possibly focussing more on individual differences.

### The principle of least effort and the owner’s influence on it

The fourth hypothesis stated that dogs always use a strategy that minimizes effort for a given context, i.e., they follow the principle of least effort (Zipf 1949). However, the predicted pattern that dogs should use relatively more high-effort strategies in close and relatively more low-effort strategies in far, irrespective of order of conditions, was not confirmed by the current results. Showing effort rather varied by time than by condition. Thus, we could not find evidence that dogs follow the principle of least effort like humans do (Zipf 1949).

It is possible, however, that an effect of the spatial set-up on dogs’ showing effort (i.e., the principle of least effort) was concealed by the interaction between dogs and owners. From the behavioral observations, it appeared that owners usually incited their dogs to show more precisely and therefore more effortfully, i.e., they did rarely accept low-effort strategies like gaze alternation, even if they would have sufficed. As the results of this study demonstrate, owners also based their decisions more often on high-effort showings. Therefore, owners might have effectively enforced a ceiling effect for showing effort which might have concealed differences in showing effort between and within conditions.

We found that owner behavior generally increased showing effort. The increase was stronger in far, where showing effort was originally predicted to be low, than in close, where showing effort was predicted to be high. Presumably, this way, the owner’s influence effectively eliminated the predicted difference in showing effort and, therefore, the hypothesized effect of the principle of least effort.

At this point, it is not completely clear whether owners did in fact influence showing effort or whether showing effort rather influenced the owners’ behavior. However, the latter case seems substantially hard to interpret and, hence, rather unlikely, as the direction of the effect would suggest that owners pushed their dogs more, the more effortfully they showed. Moreover, the explanation that the owner’s behavior generally increased showing effort fits with the activating effects of ostensive human communication described earlier. Nonetheless, this effect remains somewhat uncertain and needs further investigation, possibly also employing other operationalizations of showing effort.

### Limitations and implications for future research

Inferences about the effects of the owner’s influence on the proportion of correct showing and effort can only be made with caution since these variables were not manipulated experimentally (i.e., influence versus no influence). Future studies should aim at implementing this to get a clearer picture of the dog–human interplay.

One very interesting point this study could illuminate over and above other studies is the importance of different types of showing. Past studies have mainly focused on gazing and gaze alternation. While the current study also found this to be an important type of showing behavior, it appeared to be less important for success in the task since it converged little with the target box or the owner’s choice. In other words: it constituted only a moderately precise showing strategy from both the dog’s and the owner’s point of view. Other showing types seemed to be much more informative, especially showing involving jumping at the target box and vocalizing. Thus, in future studies, these behaviors should be investigated in addition to gaze alternation.

### Conclusion

In summary, this study confirmed that dogs use showing behavior to successfully communicate the location of a hidden object to their owner and, moreover, demonstrated that success in such a hidden-object paradigm can be truly attributed to dogs showing the target location. This study also indicated for the first time that owners can influence their dog’s showing accuracy (and thereby success) but that such influence tends to be negative rather than positive. This finding fits with previous literature that found human ostensive signals to be diffusely activating and potentially ‘accidentally deceptive’ for dogs. Moreover, owners can influence how effortfully their dogs show, generally increasing effort, especially when the task was easier. Regarding the effect of communication history, this study could neither find an effect on showing effort (strategies) nor on success in the task. In contrast, an effect of the spatial set-up was found for success, with pairs performing better when hiding places where further apart, however, not for showing effort (strategies), i.e., there was no evidence from this study that dogs followed the principle of least effort. The latter could, however, be concealed by human influence since owners enforced high-effort showing especially in the condition where effort was predicted to be low. Future research with bigger samples should focus on further illuminating the complex effects of the owner’s influence on canine showing behavior and its efficiency.

## Electronic supplementary material

Below is the link to the electronic supplementary material.

Supplementary file1 Online Resource 1 Additional information and in-depth descriptions regarding hypotheses, subjects, materials and set-up, procedure, behavioral coding and additional statistical analyses and results that are not reported in the main text, including additional graphics (PDF 938 kb)

Supplementary file2 Online Resource 2 Raw data and additional information about subjects (XLSX 919 kb)

Supplementary file3 Online Resource 3 Exemplary video of the procedure in the Close condition with the target hidden in box 4, including pretest and hiding (MP4 73369 kb)

Supplementary file4 (R 72 kb)

## Footnotes

1 In the case of two pairs (see Online Resource 2), the dogs did not pay any attention to the toy anymore as soon as the owner had left the room. Thus, it was decided that the owners could stay inside the room, but they had to cover their eyes and ears during the hiding procedure. This way, the owners remained naïve and the dogs were attentive towards the toy.

2 In 10 trials, there were problems with the procedure. Sensitivity analyses were run in which these cases were excluded (see Online Resource 1) but results did not change in regard to the predictions.

3 Again, sensitivity analyses were run excluding phases with retrieval (see Online Resource 1). Results did not change in regard to the predictions.

4 Analyses were run to determine a possible effect on performance and behavior (see Online Resource 1). The resulting patterns of success and choice rates did not reflect this uneven distribution.
