# Supplementary material for: Effect of shared information and owner behavior on showing in dogs (Canis familiaris)
Source: Anim Cogn. 2020 Jul 5;23(5):1019–34. doi: 10.1007/s10071-020-01409-9 (PMC7415761; doi:10.1007/s10071-020-01409-9)
Supplement: Supplementary file 1 — Supplementary file1 Online Resource 1 Additional information and in-depth descriptions regarding hypotheses, subjects, materials and set-up, procedure, behavioral coding and additional statistical analyses and results that are not reported in the main text, including additional graphics (PDF 938 kb) [file 10071_2020_1409_MOESM1_ESM.pdf]

Effect of Shared Information and Owner Behaviour on Showing in Dogs (*Canis familiaris*)

Melanie Henschel[*1,2], James Winters[3], Thomas F. Müller[3] and Juliane Bräuer[1,2]

[1] Department for General Psychology and Cognitive Neuroscience, Friedrich Schiller University Jena, Jena, Germany

[2] Department of Linguistic and Cultural Evolution, Max Planck Institute for the Science of Human History, 10, Kahlaische Str., 07745 Jena, Germany

[3] Minds and Traditions Research Group, Max Planck Institute for the Science of Human History, 10, Kahlaische Str., 07745 Jena, Germany

* corresponding author: henschel@shh.mpg.de

Animal Cognition

Supplementary Material

Hypotheses

H3: Dogs monitor past interactions, and this constrains future communicative behaviour (i.e. convention formation). Based on this hypothesis I predict that (a) the starting condition determines the showing strategy dogs use throughout the whole procedure, i.e. they develop a precise, high-effort strategy, starting with the Close condition and an imprecise, low-effort strategy when they start with Far. This implies that dogs should use relatively more high-effort strategies in the Close condition when it is their first session and relatively more low-effort strategies when Close comes second. Contrarily, they should use relatively more low-effort showing in the Far condition when it comes first and more high-effort strategies when Far is their second session. Thus, I predict an interaction of condition and session regarding showing effort. This has direct implications concerning performance: (b) Pairs are predicted to perform equally well in the Far condition, independent of whether it comes first or second because both a low- and a high-effort showing strategy suffice to solve the task. However, pairs are predicted to perform relatively better in the Close condition if it is completed first and relatively worse if it is completed second because only a high-effort strategy suffices to achieve success in the Close condition. Thus, I expect an interaction between condition and session regarding performance as well.

Subjects

The 32 dog-owner pairs that took part in this study were either newly recruited or chosen from the DogStudies database in which dogs and their owners who volunteer and consent for studies are logged and referenced to the studies they have participated in. Most pairs had already taken part in other studies but none of them investigated dog-owner interaction or showing behaviour. Two pairs had to be excluded during testing because of health problems of the dog, leading to a final sample size of 30 dog-owner pairs. Pairs were composed of 30 dogs and 27 owners, i.e. three owners took part with two dogs each. Tests

took place at the dog laboratory of the Max Planck Institute for the Science of Human History in Jena between June and November 2018. After completing the experiment, they received a dog toy or dog treats as an expense allowance. Additionally, after data collection was finished, owners had access to all videos of themselves and their dogs filmed during their stay at the lab.

Materials and set-up

In the test room, four shoe boxes (8 cm x 15 cm x 20 cm) were set up which constituted the four possible hiding places. All boxes were painted black to look the same, thus, any preferences due to appearance of the boxes could be excluded. In the test room the boxes were attached to the windowsills with hook and loop fastener. Each box was approximately 3.5 m away from the owner and approximately 1.1 m above the ground. In the test room the boxes were attached to the windowsills with hook and loop fastener. Above the boxes, a short version of the instructions was stuck to the wall as a reminder for the owner during the test (Figure SI1). On the chair for the owners in the middle of the room, a questionnaire (see Figure SI2) was placed on which owners had to check the supposed target box. Each trial was filmed by two cameras, one was set up above the boxes in front of the owner, the other was positioned behind the owner. Two experimenters guided the owners and their dogs through the experiment. Owners were instructed beforehand to bring their dog's

favourite toy. If the owner forgot the toy or it was too big for the boxes, a substitute toy from the laboratory was offered, provided that the dog liked to play with the substitute toy.

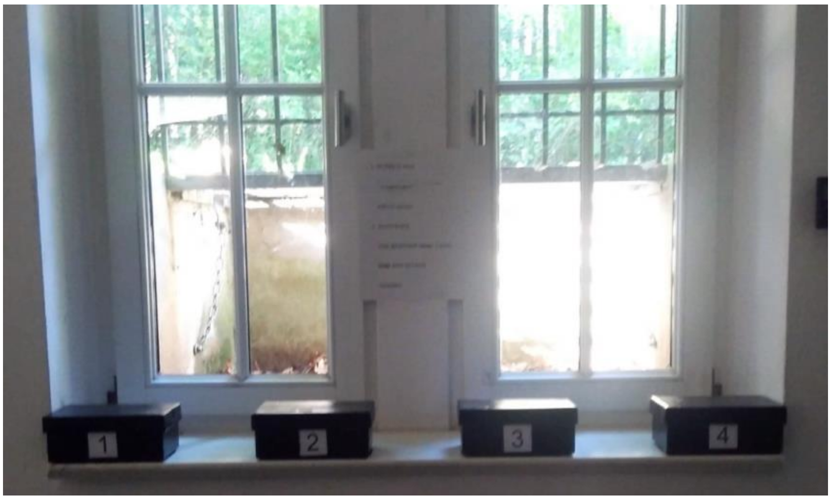

Figure SI1. Exemplary set-up of the boxes, depicted here is the Close condition, including the ‘cheat sheet’ for the owners.

Phase 1: In welcher Box vermuten Sie das Spielzeug? Bitte kreuzen Sie die Zahl an, welche auf der entsprechenden Box steht.

<u>Phase1</u>

Durchgang Nr. 1:

□ 1 □ 2 □ 3 □ 4

Durchgang Nr. 2

□ 1 □ 2 □ 3 □ 4

Durchgang Nr. 3

□ 1 □ 2 □ 3 □ 4

Durchgang Nr. 4

□ 1 □ 2 □ 3 □ 4

Figure SI2. Questionnaire for choices in phase 1, handed out once per session. The title translates to: “Phase 1: In which box do you assume the toy? Please check the number which is written on the corresponding box.” The captions for each line of boxes to be checked translates to: “Trial number” 1 to 4.

Procedure

Before data collection began, the set-up was piloted with two dogs and their owners. Each condition was tested with only one pair since the purpose here was just to test set-up and procedure and to train the procedure for the experimenters. Accordingly, these two pairs were not included in the final sample. After some improvements the actual test was conducted.

In 10 cases, the owners did not follow the instructions correctly and gave the toy to their dog although it had shown the wrong box or the dog got hold of the toy while the experimenter showed the correct location. Included in these "problem cases" was also one case in which the experimenter said the target box out loud and the owner might have heard through the door of the test room, one case in which the experimenter began hiding before the door of the test room was fully closed which the owner might have seen and one case in which the toy was too big and pushed up the lid of the box which was only corrected after the first trial. Sensitivity analyses were run in which these cases, either on trial or pair basis, were excluded (see Online Resource 3). Results did not change in regard to the predictions.

Behavioural coding

Gaze direction was coded as eye gaze in the narrower sense (i.e. an imaginary straight line starting vertically from the centre of the pupil leading to the perceived target) whenever the dog's eyes were visible and otherwise via direction of the snout. Movements were coded via direction of steps, not body curvature, head direction or perceived intended direction of movement, to reduce coder bias and avoid confounding with gazing.

How often dogs spent time near each box was coded via position of the dog's head. For this variable we defined near zones for each box (see Figure SI3) which are of different size. Four identical areas defined for each condition would have been more standardized but would not have met the real situation from the point of view of the owners. If for example the dog stood in the middle between door 2 and box 1 in Close, in case of identically sized zones, the dog would not have been in the near zone of any box. But de facto the box nearest to the

dog would still be box number 1. Indeed, during piloting owners reported taking this into account. Therefore, the near zones were defined accordingly and a possible effect of position of boxes on performance and choice was analysed statistically (see section Statistical analysis and results of the paper).

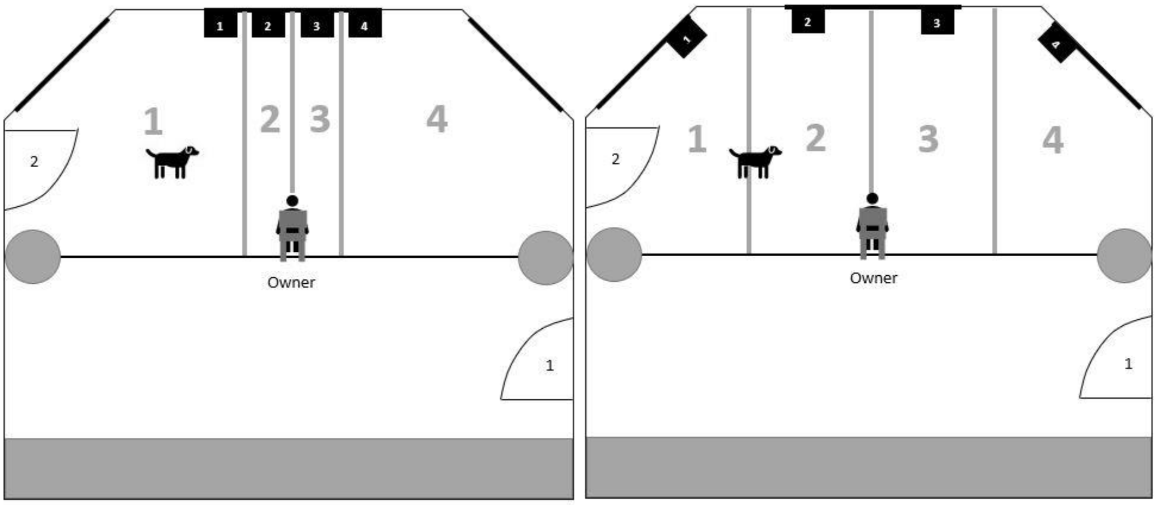

Figure SI3. Near zones in the Close condition (left) and the Far condition (right). In the left picture, the dog would be coded to be located near box 1, in the right picture near box 2.

Jumping/standing upright was coded if the dog's front legs were lifted from the ground and pointed towards the boxes. Again, position of the dog's head determined which box was coded. Vocalizations included all occurring barks, howls and whimpers. Opening a box was coded as soon as the lid of a box was not in place anymore, i.e. even if the lid fell back into place after the dog's manipulation. Retrieval was coded as soon as the toy was touched by the dog's mouth and moved outside of the box.

To specify showing behaviours, the above-mentioned behaviours were divided into directional components and attention-getting components. Directional components comprised all behaviours directed at one specific box, i.e. gazing at a box, moving towards a box, spending time near a box, jumping/standing upright in front of a box and opening a box. Retrieving the toy did indeed address one specific box as well, however, it constitutes a choice on the part of the dog rather than giving the owner directions towards the toy. Attention-getting components included all behaviours that enabled the dog to capture the

owner's attention, i.e. gazing at the owner, moving towards the owner and vocalizing. All 15 possible combinations of these components form the showing behaviours analysed in this study. Multiple different types of showing could occur simultaneously while still being counted separately, e.g. moving towards a box followed by moving towards the owner was usually accompanied by gazing at the respective box followed by gazing at the owner. This would be counted as two different instances of showing. Flowcharts depicting the algorithm employed to calculate showings and an example of the generated output can be seen in Figures SI4 to SI7.

For owners, three different behaviours were coded: non-verbal prompting, talking and calling the dog by its name. The variable non-verbal prompting included owners' gazing at the dog, gazing at the boxes (i.e. one specific box or the general direction of the boxes), pointing at the boxes, nodding in the direction of the boxes, showing empty hands, shrugging and approaching the boxes. Talking was coded for any utterances the owner made (except calling the dog's name), i.e. including laughing, sneezing, coughing, clicking the tongue etc. This also included verbal prompting, such as "Show me the toy!" or reinforcement in the form of praising the dog. Both of these variables are very broadly defined since, for an explorative analysis of the interaction of owner and dog, the variables should cover a wide range of possibly influential behaviours. Calling the dog by its name was coded whenever the owner said the dog's name out loud. This included obvious nickname versions of the dog's name (e.g. Sue for Susi) but no other kinds of nicknames that were given (e.g. honey).

Results

Overall performance

The same results as in the main analysis were obtained if instead an individual criterion (five or more correct choices per phase, binomial test: $p = .027$) was applied. In both phases, a significant number of pairs reached this criterion: 13 pairs in phase 1 (binomial test: $p = .022$) and 15 in phase 2 (binomial test: $p = .003$).

Effect of condition on showing types

No significant differences were found for gazing at box plus moving towards owner (Far: *M* = 1.07, *SD* = 1.27, Close: *M* = 0.91, *SD* = 1.26, *t*[239] = -1.60, *p* = .110, Cohen's *d* = 0.13, 95% CI [-0.05, 0.30]), gazing at box plus vocalizing (Far: *M* = 0.34, *SD* = 1.36, Close: *M* = 0.57, *SD* = 2.13, *t*[239] = 1.97, *p* = .050, Cohen's *d* = 0.13, 95% CI [-0.05, 0.31]), moving towards box plus vocalizing (Far: *M* = 0.27, *SD* = 0.87, Close: *M* = 0.45, *SD* = 1.59, *t*[239] = 1.91, *p* = .058, Cohen's *d* = 0.14, 95% CI [-0.04, 0.32]), spending time near box plus moving towards owner (Far: *M* = 7.47, *SD* = 6.06, Close: *M* = 7.57, *SD* = 6.84, *t*[239] = 0.30, *p* = .768, Cohen's *d* = 0.01, 95% CI [-0.16, 0.19]), jumping/standing upright at box plus gazing at owner (Far: *M* = 0.55, *SD* = 1.06, Close: *M* = 0.59, *SD* = 1.31, *t*[239] = -0.21, *p* = .835, Cohen's *d* = 0.03, 95% CI [-0.15, 0.21]), jumping/standing upright at box plus moving towards owner (Far: *M* = 0.40, *SD* = 0.74, Close: *M* = 0.33, *SD* = 0.85, *t*[239] = -1.08, *p* = .282, Cohen's *d* = 0.08, 95% CI [-0.10, 0.26]), jumping/standing upright at box plus vocalizing (Far: *M* = 0.08, *SD* = 0.31, Close: *M* = 0.15, *SD* = 0.56, *t*[239] = 1.90, *p* = .059, Cohen's *d* = 0.15, 95% CI [-0.03, 0.33]), opening box plus move towards owner (Far: *M* = 0.03, *SD* = 0.17, Close: *M* = 0.02, *SD* = 0.18, *t*[239] = -0.26, *p* = .797, Cohen's *d* = 0.02, 95% CI [-0.16, 0.20]) and opening box plus vocalizing (Far: *M* = 0.01, *SD* = 0.09, Close: *M* = 0.01, *SD* = 0.09, *t*[239] = 0.00, *p* = 1.00, Cohen's *d* = 0.00, 95% CI [-0.18, 0.18]). Opening box plus gazing at owner was not used at all.

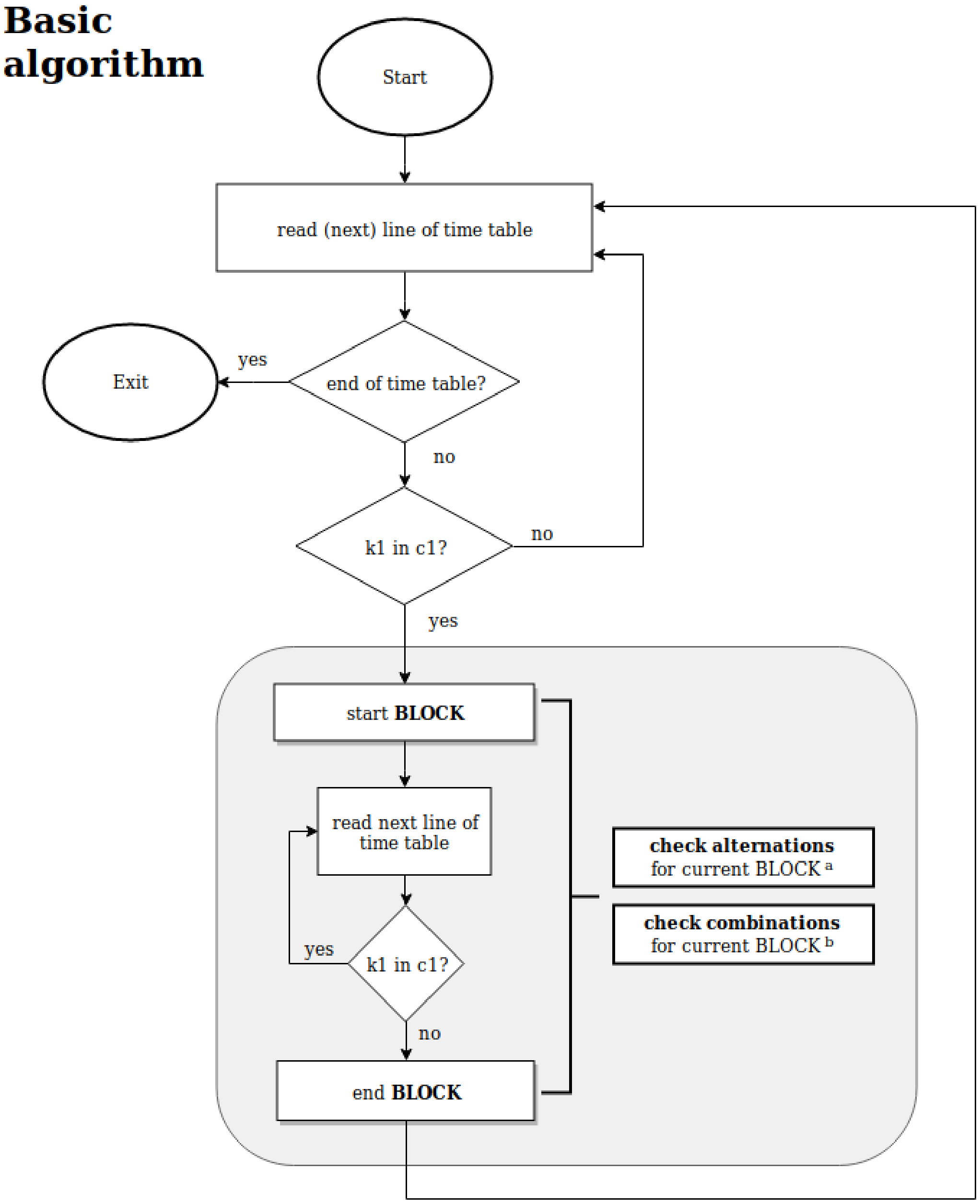

Figure SI4. The algorithm goes through the time table (Solomon Coder output) line by line, searches for BLOCKs (continuous behaviour without interruption) of the focal directional component (k1) and then checks each BLOCK for alternations and combinations with the respective attention-getting component (k2). The depicted process is run once for each predefined type of showing. Thus, only two columns are compared at a time, one containing k1 (c1) and one containing k2 (c2). Apart from k1 and k2, each column always contains the respective other variables of the same type as well, e.g. if k1 is gazing at box 1, c1 also contains gazing at all other boxes and the owner.

[a] see Figure SI5

[b] see Figure SI6

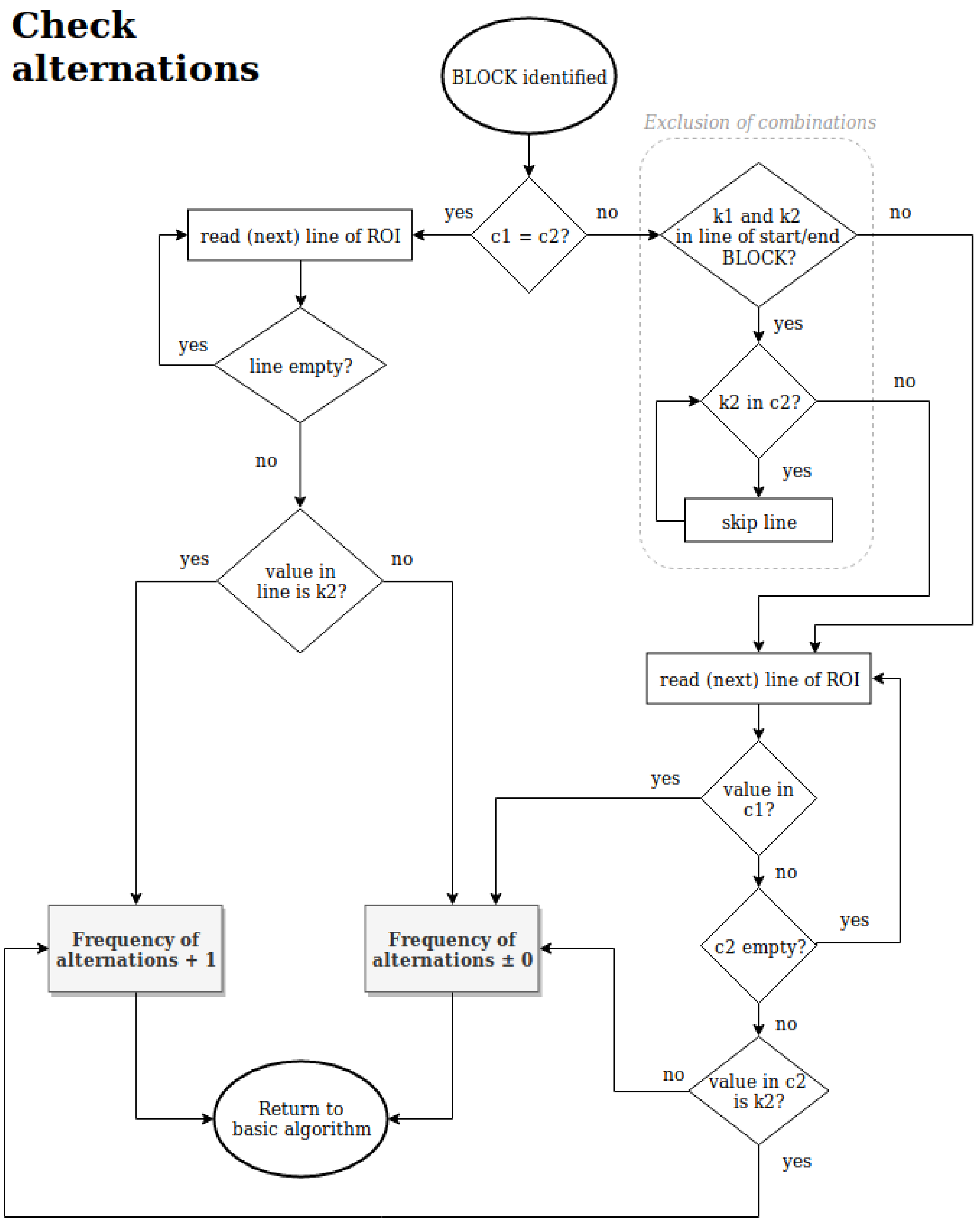

Figure SI5. Alternations are checked for a given BLOCK (continuous behaviour without interruption) of k1 (directional component of the respective showing), i.e. k2 (attention-getting component of the respective showing) is searched within two seconds before (ROI_1) and two seconds after the BLOCK (ROI_2). Within ROI_1 the algorithm goes backwards through the lines, within ROI_2 forward. The column containing k1 is c1, c2 contains k2. C1 = c2 is the case if both k1 and k2 are of the same type, e.g. k1 = gaze at box 1 and k2 = gaze at owner.

ROI_1 =]start BLOCK, start BLOCK – (2s / 0.2s)]

ROI_2 =]end BLOCK, end BLOCK + (2s / 0.2s)]

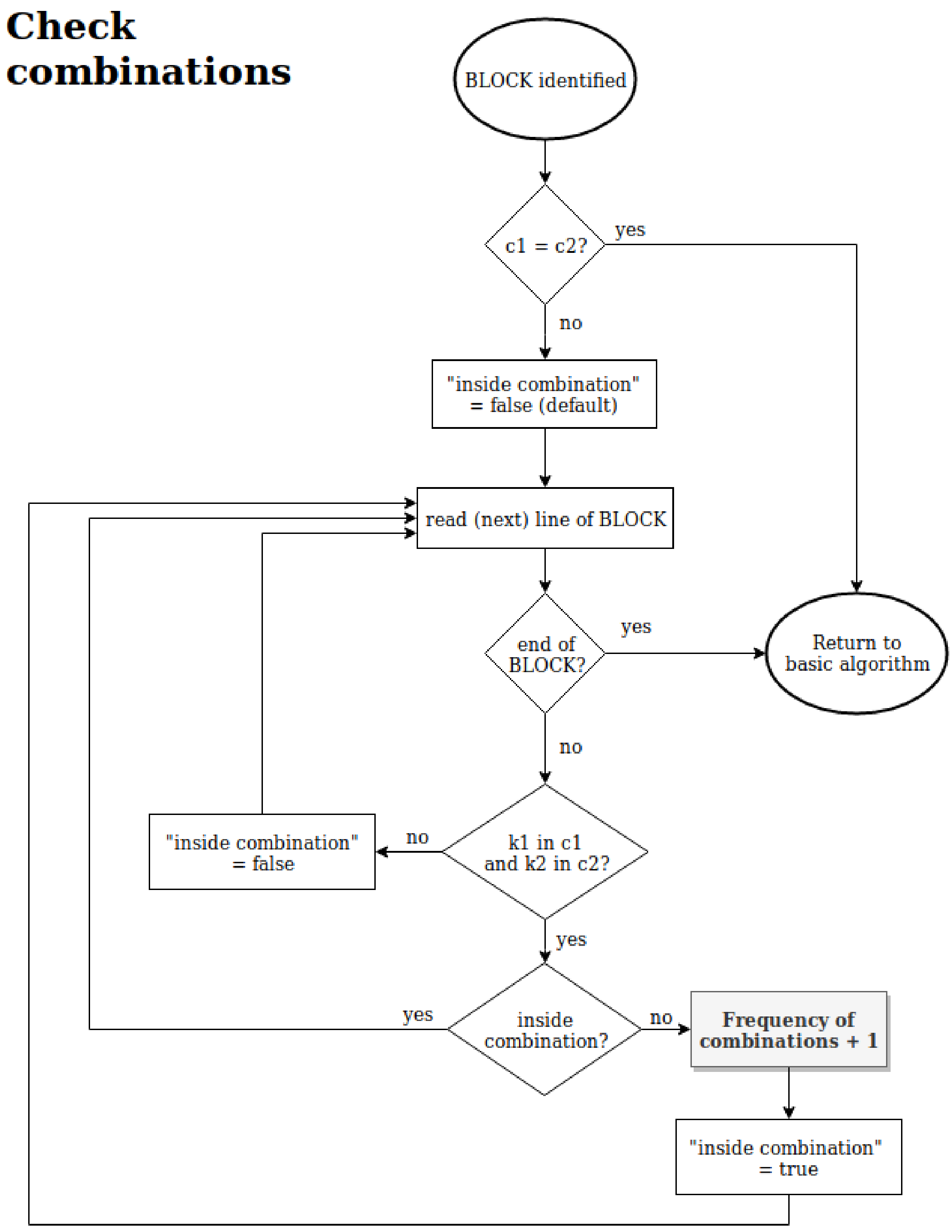

Figure SI6. Combinations are checked for a given BLOCK (continuous behaviour without interruption) of k1 (directional component of the respective showing), i.e. overlaps of k1 and blocks of k2 (attention-getting component for the respective showing) are searched. The column containing k1 is c1, c2 contains k2.

```
                                      move_1    <=> gaze_owner
-----------------------------------------------------------------------------------------------------
A? line,time     | moving                           | gaze_direction                   | phase
-----------------------------------------------------------------------------------------------------
    377, 075.40 |                                   | 'gaze_owner'                     | 2
    378, 075.60 |                                   | 'gaze_owner'                     | 2
    379, 075.80 |                                   | 'gaze_owner'                     | 2
    380, 076.00 |                                   | 'gaze_owner'                     | 2
    381, 076.20 |                                   | 'gaze_owner'                     | 2
    382, 076.40 |                                   | 'gaze_owner'                     | 2
    383, 076.60 |                                   | 'gaze_owner'                     | 2
    384, 076.80 | 'move_owner'                      | 'gaze_owner'                     | 2
    385, 077.00 | 'move_owner'                      | 'gaze_owner'                     | 2
    386, 077.20 | 'move_owner'                      | 'gaze_owner'                     | 2
    387, 077.40 | 'move_owner'                      | 'gaze_owner'                     | 2
>C  388, 077.60 | 'move_1'                          | 'gaze_owner'                     | 2
>   389, 077.80 | 'move_1'                          | 'gaze_owner'                     | 2
>   390, 078.00 | 'move_1'                          | 'gaze_4'                         | 2
>   391, 078.20 | 'move_1'                          |                                  | 2
>   392, 078.40 | 'move_1'                          | 'gaze_1'                         | 2
>   393, 078.60 | 'move_1'                          | 'gaze_1'                         | 2
    394, 078.80 | 'move_1'                          |                                  | 2
 A  395, 079.00 |                                   | 'gaze_owner'                     | 2
    396, 079.20 |                                   | 'gaze_owner'                     | 2
    397, 079.40 |                                   | 'gaze_owner'                     | 2
    398, 079.60 | 'move_owner'                      | 'gaze_owner'                     | 2
    399, 079.80 | 'move_owner'                      | 'gaze_owner'                     | 2
    400, 080.00 | 'move_owner'                      | 'gaze_owner'                     | 2
    401, 080.20 |                                   | 'gaze_owner'                     | 2
    402, 080.40 |                                   | 'gaze_owner'                     | 2
    403, 080.60 | 'move_4'                          | 'gaze_owner'                     | 2
    404, 080.80 | 'move_4'                          | 'gaze_owner'                     | 2
-----------------------------------------------------------------------------------------------------
Found   1 Alternations and   1 Combined Frequencies
```

Figure SI7. Exemplary Python output depicting the calculation of alternations (A) and combinations (C) of directional components (here: moving towards box 1) and attention-getting components (here: gazing at the owner). Each change from directional to attention-getting-component or vice versa is counted as one instance of showing. The focal behavioural unit is marked with ‘>’.

Appendix A

Results of sensitivity analyses for analyses regarding overall performance, and the effects of correct showing, condition, session, trial and phase on success

Table A1

Results of t-tests of mean difference between phases and t-tests against chance for each phase for data sets excluding problem trials, problem pairs and rows with retrieval, respectively

**Test**	***M***	***SD***	***t***	***df***	***p***	**Cohen's *d***	**95 % CI**
				Phase 1 vs. 2			
Problem trials[a] excl.			**-1.37**	**22**	**.184**	**0.14**	**[-0.37, 0.64]**
Problem pairs[b] excl.			**-1.37**	**22**	**.184**	**0.14**	**[-0.37, 0.64]**
Retrieval[c] excl.			**-1.74**	**23**	**.096**	**0.42**	**[-0.10, 0.93]**
				Phase1			
Problem trials[a] excl.	*55.43*	*27.91*	*9.48*	*22*	*< .001*	*1.98*	*[1.08, 2.85]*
Problem pairs[b] excl.	*55.43*	*27.91*	*9.48*	*22*	*< .001*	*1.98*	*[1.08, 2.85]*
Retrieval[c] excl.	*46.35*	*21.01*	*10.75*	*23*	*< .001*	*2.19*	*[1.27, 3.10]*
				Phase 2			
Problem trials[a] excl.	*59.24*	*27.75*	*10.19*	*22*	*< .001*	*2.13*	*[1.21, 3.02]*
Problem pairs[b] excl.	*59.24*	*27.75*	*10.19*	*22*	*< .001*	*2.13*	*[1.21, 3.02]*
Retrieval[c] excl.	*55.56*	*22.82*	*12.59*	*26*	*< .001*	*2.42*	*[1.46, 3.37]*

Note. Significant results are printed in italics. Significances differing from the main analyses are printed in bold.

[a] $n = 10$, corresponds to 20 data rows

[b] $n = 7$, corresponds to 112 data rows

[c] $n = 28$

Table A2

Results of model comparisons for data sets excluding problem trials, problem pairs and rows with retrieval, respectively

**Comparison**	**AIC**	**χ^2**	***df***	***p***
Success ~ correct showing + session/trial/phase				
Problem trials excluded (*n* = 10)				
Null model	562.09			
+ session/trial/phase	**561.68**	**10.41**	**5**	**.064**
+ correct showing	*513.03*	*50.65*	*1*	*< .001*
Problem pairs excluded (*n* = 7)				
Null model	430.61			
+ session/trial/phase	**433.60**	**7.01**	**5**	**.219**
+ correct showing	*402.59*	*33.01*	*1*	*< .001*
Retrieval excluded (*n* = 26)				
Null model	578.72			
+ session/trial/phase	*574.37*	*14.35*	*5*	*.014*
+ correct showing	*518.71*	*57.66*	*1*	*< .001*
Success ~ condition + session/trial/phase				
Problem trials excluded (*n* = 10)				
Null model	580.69			
+ session/trial/phase	*578.95*	*11.73*	*5*	*.039*
+ condition	*565.13*	*15.83*	*1*	*< .001*
+ condition:session/trial/phase	571.78	3.35	5	.647
Problem pairs excluded (*n* = 7)				
Null model	448.33			
+ session/trial/phase	**450.39**	**7.93**	**5**	**.160**
+ condition	*439.54*	*12.85*	*1*	*< .001*
+ condition:session/trial/phase	447.52	2.03	5	.845
Retrieval excluded (*n* = 28)				
Null model	600.74			
+ session/trial/phase	*595.87*	*14.88*	*5*	*.011*

(continued)

Table A2

Results of model comparisons for data sets excluding problem trials, problem pairs and retrieval, respectively (continued)

**Comparison**	**AIC**	**χ^2**	***df***	***p***
+ condition	*582.43*	*15.43*	*1*	*< .001*
+ condition:session/trial/phase	587.57	4.87	5	.432
Showing effort ~ session/trial/phase				
Problem trials excluded (*n* = 10)				
Null model	-202.31			
+ session/trial/phase	*-277.30*	*84.98*	*5*	*< .001*
+ condition	-276.13	0.84	1	.360
+ condition:session/trial/phase	-266.76	0.63	5	.987
Problem pairs excluded (*n* = 7)				
Null model	-141.37			
+ session/trial/phase	*-198.48*	*67.11*	*5*	*< .001*
+ condition	-196.57	0.09	1	.761
+ condition:session/trial/phase	-189.16	2.59	5	.763
Retrieval excluded (*n* = 26)				
Null model	-206.41			
+ session/trial/phase	*-286.39*	*89.99*	*5*	*< .001*
+ condition	-286.37	1.98	1	.159
+ condition:session/trial/phase	-277.92	1.54	5	.908

Note. The null model only contained pair ID as random intercept. Sample sizes for problem trials, problem pairs and retrievals are indicated in different units. One data row corresponds to one phase within a trial of a given session for a given pair, i.e. sample sizes for problem pairs have to be multiplied by 16 and problem trials by 2 to receive the amount of involved data rows. Significant results are printed in italics. Significances differing from the main analyses are printed in bold.

Table A3

Random effects of final the GLMMs and LMM for data sets excluding problem trials, problem pairs and retrieval, respectively

**Random intercepts**[a]	**Variance**	***SD***
Success ~ correct showing + session/trial/phase		
Problem trials excluded (*n* = 10)	1.01	1.01
Problem pairs excluded (*n* = 7)	0.52	0.72
Retrieval excluded (*n* = 26)	1.38	1.17
Success ~ condition + session/trial/phase		
Problem trials excluded (*n* = 10)	1.39	1.18
Problem pairs excluded (*n* = 7)	2.05	1.43
Retrieval excluded (*n* = 28)	0.85	0.92
Showing effort ~ session/trial/phase		
Problem trials excluded (*n* = 10)	0.00	0.07
Problem pairs excluded (*n* = 7)	0.00	0.06
Retrieval excluded (*n* = 26)	0.01	0.07

Note. Sample sizes for problem trials, problem pairs and retrievals are indicated in different units. One data row corresponds to one phase within a trial of a given session for a given pair, i.e. sample sizes for problem pairs have to be multiplied by 16 and problem trials by 2 to receive the amount of involved data rows.

[a] In all models the random intercept was pair ID.

Table A4

Fixed effects of final GLMM for the effect of correct showing on success with data sets excluding problem trials, problem pairs and retrieval, respectively

**Fixed effects**	**β**	***SE***	***z***	***p***
Problem trials excluded (*n* = 10)				
(Intercept)	-0.46	0.53	-0.87	.382
correct showing	*6.73*	*1.05*	*6.39*	*< .001*
session	-0.70	0.58	-1.20	0.229
session1:trial	*-0.41*	*0.16*	*-2.59*	*.010*
session2:trial	-0.12	0.16	-0.77	.440
session1:trial:phase	0.13	0.12	1.14	.255
session2:trial:phase	0.11	0.12	0.93	.351
Problem pairs excluded (*n* = 7)				
(Intercept)	-0.39	0.60	-0.65	.517
correct showing	*5.82*	*1.10*	*5.28*	*< .001*
session	-0.54	0.65	-0.83	.407
session1:trial	*-0.37*	*0.18*	*-2.10*	*.036*
session2:trial	-0.09	0.18	-0.52	.602
session1:trial:phase	0.12	0.14	0.86	.391
session2:trial:phase	0.07	0.14	0.48	.631
Retrieval excluded (*n* = 26)				
(Intercept)	-0.69	0.49	-1.39	.164
correct showing	*7.01*	*1.04*	*6.75*	*< .001*
session	-0.52	0.56	-0.94	.345
session1:trial	*-0.47*	*0.16*	*-3.01*	*.003*
session2:trial	-0.22	0.16	-1.41	.159
session1:trial:phase	0.17	0.12	1.45	.147
session2:trial:phase	0.18	0.12	1.53	.127

Note. Sample sizes for problem trials, problem pairs and retrievals are indicated in different units. One data row corresponds to one phase within a trial of a given session for a given pair, i.e. sample sizes for problem pairs have to be multiplied by 16 and problem trials by 2 to receive the amount of involved data rows.
Wald's *z*-score = β / *SE*, significant results are printed in italics. Significances differing from the main results are printed in bold.

Table A5

Fixed effects of final GLMM for the effect of condition and session/trial/phase on success with data sets excluding problem trials, problem pairs and retrieval, respectively

**Fixed effects**	**β**	***SE***	***z***	***p***
Problem trials excluded (*n* = 10)				
(Intercept)	*1.15*	*0.46*	*2.50*	*.013*
condition	*0.87*	*0.22*	*3.93*	*< .001*
session	-1.00	0.55	-1.82	.068
session1:trial	*-0.48*	*0.15*	*-3.11*	*.002*
session2:trial	-0.15	0.15	-1.05	.296
session1:trial:phase	0.11	0.11	0.99	.324
session2:trial:phase	0.14	0.11	1.31	.191
Problem pairs excluded (*n* = 7)				
(Intercept)	*1.14*	*0.55*	*2.08*	*.037*
condition	*0.91*	*0.26*	*3.54*	*< .001*
session	-1.05	0.62	-1.69	.090
session1:trial	*-0.47*	*0.18*	*-2.68*	*.007*
session2:trial	-0.09	0.17	-0.51	.610
session1:trial:phase	0.08	0.13	0.59	.554
session2:trial:phase	0.10	0.13	0.77	.440
Retrieval excluded (*n* = 28)				
(Intercept)	*0.92*	*0.42*	*2.16*	*.030*
condition	*0.82*	*0.21*	*3.88*	*< .001*
session	-0.77	0.52	-1.47	.141
session1:trial	*-0.49*	*0.15*	*-3.28*	*.001*
session2:trial	-0.23	0.15	-1.54	.123
session1:trial:phase	0.13	0.11	1.16	.244
session2:trial:phase	0.20	0.11	1.83	.067

Note. Sample sizes for problem trials, problem pairs and retrievals are indicated in different units. One data row corresponds to one phase within a trial of a given session for a given pair, i.e. sample sizes for problem pairs have to be multiplied by 16 and problem trials by 2 to receive the amount of involved data rows.
Significant results are printed in italics. Significances differing from the main analyses are printed in bold.

Table A6

Fixed effects of final LMM for the effect of session/trial/phase on showing effort with data sets excluding problem trials, problem pairs and retrieval, respectively

**Fixed effects**	**β**	***SE***	***t***	***df***	***p***
Problem trials excluded (*n* = 10)					
(Intercept)	*0.39*	*0.03*	*12.65*	*302.87*	*< .001*
session	0.00	0.04	0.01	397.45	.947
session1:trial	0.01	0.01	1.06	398.18	.292
session2:trial	0.00	0.01	-0.14	396.84	.887
session1:trial:phase	*-0.05*	*0.01*	*-6.56*	*400.00*	*< .001*
session2:trial:phase	*-0.05*	*0.01*	*-6.36*	*400.34*	*< .001*
Problem pairs excluded (*n* = 7)					
(Intercept)	*0.38*	*0.03*	*11.01*	*255.92*	*< .001*
session	0.02	0.05	0.49	312.87	.628
session1:trial	0.02	0.01	1.41	312.83	.160
session2:trial	-0.01	0.01	-0.42	312.88	.673
session1:trial:phase	*-0.06*	*0.01*	*-6.34*	*316.82*	*< .001*
session2:trial:phase	*-0.05*	*0.01*	*-5.14*	*317.32*	*< .001*
Retrieval excluded (*n* = 26)					
(Intercept)	*0.37*	*0.03*	*12.18*	*271.64*	*< .001*
session	0.02	0.04	0.44	394.88	.662
session1:trial	0.02	0.01	1.78	394.55	.076
session2:trial	0.00	0.01	0.27	392.75	.791
session1:trial:phase	*-0.05*	*0.01*	*-6.55*	*396.32*	*< .001*
session2:trial:phase	*-0.06*	*.001*	*-6.87*	*395.06*	*< .001*

Note. Sample sizes for problem trials, problem pairs and retrievals are indicated in different units. One data row corresponds to one phase within a trial of a given session for a given pair, i.e. sample sizes for problem pairs have to be multiplied by 16 and problem trials by 2 to receive the amount of involved data rows.
Significant results are printed in italics.

Appendix B

Differences between boxes

Table B1

Pairwise comparisons of success rates and choices for each box per pair and trial

	***p*-values**					
	**Success rates**			**Choices**		
	**Box 1**	**Box 2**	**Box 3**	**Box 1**	**Box 2**	**Box 3**
**Box 2**	.764			1.00		
**Box 3**	.024 *	1.00		.960	1.00	
**Box 4**	.688	1.00	1.00	1.00	1.00	1.00

Note. Significance code: $p < .05$ *

Table B2

Inference statistics for choices of each box per pair and trial

**Box**	***M***	***SD***	***t*[29]**	***p***	**Cohen's *d***	**95 % CI**
**1**	0.23	0.12	-0.84	.411	0.15	[-0.57, 0.87]
**2**	0.24	0.12	-0.68	.504	0.12	[-0.59, 0.84]
**3**	0.28	0.12	-1.12	.272	0.20	[-0.52, 0.92]
**4**	0.24	0.12	-0.29	.771	0.05	[-0.66, 0.77]

Appendix C

Spearman rank order correlation coefficients (with significances) to assess inter-coder reliability for frequency and duration of gazes at each box and the owner, movements towards each box and the owner, time spent near each box, and jumping/standing upright at each box, and frequency of opening each box.

	**Box 1**		**Box 2**		**Box 3**		**Box 4**		**Owner**	
	r_s	*p*	r_s	*p*	r_s	*p*	r_s	*p*	r_s	*p*
					Frequencies					
Gaze	.60	< .001	.80	< .001	.71	< .001	.70	< .001	.90	< .001
Move	.75	< .001	.79	< .001	.69	< .001	.80	< .001	.88	< .001
Time	.98	< .001	.96	< .001	.97	< .001	.96	< .001	-	-
Jump	1.00	< .001	.99	< .001	.94	< .001	1.00	< .001	-	-
Open	1.00	< .001	.87	< .001	.88	< .001	.91	< .001	-	-
					Durations					
Gaze	.69	< .001	.89	< .001	.80	< .001	.77	< .001	.95	< .001
Move	.82	< .001	.79	< .001	.76	< .001	.83	< .001	.90	< .001
Time	.99	< .001	.96	< .001	.93	< .001	.97	< .001	-	-
Jump	1.00	< .001	1.00	< .001	.95	< .001	1.00	< .001	-	-

Appendix D

Result summary of all GLMMs and LMMs calculated for model comparisons including final models in for analyses regarding the effects of correct showing, condition, session, trial and phase on success

Table D1

Result summary of the GLMMs calculated for model comparisons regarding the effect of correct showing on success

**Random intercepts**[a]	**Variance**		***SD***	
Null model	0.90		0.95	
+ session/trial/phase	0.96		0.98	
+ correct showing (final model)	0.86		0.93	
**Fixed effects**	**β**	***SE***	***z***	***p***
+ session/trial/phase				
(Intercept)	*1.32*	*0.42*	*3.17*	*.002*
S	-0.70	0.51	-1.36	.175
S1:T	*-0.43*	*0.14*	*-3.00*	*.003*
S2:T	-0.17	0.13	-1.24	.217
S1:T:P	0.06	0.11	0.56	.576
S2:T:P	0.10	0.11	0.98	.329
+ correct showing (final model)				
(Intercept)	-0.59	0.51	-1.16	.244
correct showing	*6.81*	*1.03*	*6.62*	*<.001*
session	-0.52	0.56	-0.94	.347
session1:trial	*-0.40*	*0.15*	*-2.64*	*.008*
session2:trial	-0.15	0.15	-1.00	.316
session1:trial:phase	0.11	0.11	0.99	.324
session2:trial:phase	0.12	0.12	1.03	.304

Note. Wald's *z*-score = β / *SE*, S = session, C = condition, T = trial, P = phase, significant coefficients are printed in italics.

[a] In all models the random intercept was pair ID.

Table D2

Result summary of the GLMMs calculated for model comparisons regarding the effect of condition, session/trial/phase and their interaction on success

**Random intercepts**[a]	**Variance**		***SD***	
Null model	1.12		1.06	
+ session/trial/phase	1.20		1.09	
+ condition (final model)	1.30		1.14	
+ session/trial/phase:condition	1.33		1.15	
**Fixed effects**	**β**	***SE***	***z***	***p***
+ session/trial/phase				
(Intercept)	*1.39*	*0.42*	*3.30*	*< .001*
S	-0.78	0.51	-1.52	.128
S1:T	*-0.45*	*0.14*	*-3.15*	*.002*
S2:T	-0.17	0.14	-1.20	.232
S1:T:P	0.09	0.11	0.85	.395
S2:T:P	0.13	0.11	1.27	.205
+ condition (final model)				
(Intercept)	*1.06*	*0.44*	*2.40*	*.016*
condition	*0.78*	*0.21*	*3.71*	*<.001*
session	-0.81	0.52	-1.56	.118
session1:trial	*-0.47*	*0.14*	*-3.21*	*.001*
session2:trial	-0.17	0.14	-1.21	.226
session1:trial:phase	0.09	0.11	0.87	.385
session2:trial:phase	0.14	0.11	1.28	.200
+ session/trial/phase:condition				
(Intercept)	0.90	0.60	1.51	.130
C	1.12	0.87	1.28	.202
S	-0.28	0.84	-0.33	.739
C:S	-1.10	1.35	-0.81	.417
CClose:S1:T	*-0.41*	*0.21*	*-2.02*	*.044*
CFar:S1:T	*-0.53*	*0.21*	*-2.51*	*.012*
CClose:S2:T	-0.35	0.20	-1.70	.089

(continued)

Table D2

Result summary of the GLMMs calculated for model comparisons regarding the effect of condition, session/trial/phase and their interaction on success (continued)

**Fixed effects**	**β**	***SE***	***z***	***p***
CFar:S2:T	0.01	0.20	0.03	.977
CClose:S1:T:P	0.04	0.15	0.23	.817
CFar:S1:T:P	0.15	0.15	0.98	.325
CClose:S2:T:P	0.25	0.15	1.63	.102
CFar:S2:T:P	0.02	0.15	0.15	.878

Note. Wald's *z*-score = β / *SE*, S = session, C = condition, T = trial, P = phase, significant coefficients are printed in italics.

[a] In all models the random intercept was pair ID.

Table D3

Result summary of the LMMs calculated for model comparisons regarding the effect of condition, session/trial/phase and their interaction on showing effort

**Fixed effects**[a]	**β**	***SE***	***t***	***df***	***p***
+ session/trial/phase (final model)					
(Intercept)	*0.39*	*0.03*	*13.02*	*293.28*	*< .001*
S	0.01	0.04	0.26	416.92	.792
S1:T	0.01	0.01	1.06	416.89	.290
S2:T	0.00	0.01	-0.40	416.88	.688
S1:T:P	*-0.05*	*0.01*	*-6.43*	*420.37*	*< .001*
S2:T:P	*-0.05*	*0.01*	*-6.65*	*420.75*	*< .001*
+ condition					
(Intercept)	*0.38*	*0.03*	*12.26*	*312.27*	*< .001*
C	0.02	0.02	1.10	415.53	.271
S	0.01	0.04	0.28	415.91	.782
S1:T	0.01	0.01	1.08	415.88	.283
S2:T	0.00	0.01	-0.40	415.87	.687
S1:T:P	*-0.05*	*0.01*	*-6.43*	*419.36*	*< .001*
S2:T:P	*-0.05*	*0.01*	*-6.67*	*419.76*	*< .001*
+ session/trial/phase:condition					
(Intercept)	*0.38*	*0.04*	*8.93*	*286.02*	*< .001*
C	0.01	0.06	0.17	281.08	.865
S	0.03	0.06	0.42	286.93	.674
C:S	-0.03	0.09	-0.32	141.14	.750
CClose:S1:T	0.01	0.02	0.45	411.96	.653
CFar:S1:T	0.02	0.01	1.06	411.54	.289
CClose:S2:T	-0.01	0.02	-0.70	411.90	.487
CFar:S2:T	0.00	0.01	0.12	411.68	.903
CClose:S1:T:P	*-0.05*	*0.01*	*-4.36*	*415.98*	*< .001*
CFar:S1:T:P	*-0.05*	*0.01*	*-4.69*	*414.39*	*< .001*
CClose:S2:T:P	*-0.05*	*0.01*	*-4.41*	*416.20*	*< .001*
CFar:S2:T:P	*-0.06*	*0.01*	*-4.98*	*414.95*	*< .001*

Note. S = session, C = condition, T = trial, P = phase, significant coefficients are printed in italics.

[a] In all models the random intercept was pair ID with a variance of 0.00 and a standard deviation of 0.07.

Appendix E

Results for analyses regarding the effects of correct showing, condition, session, trial and phase on success adjusted for outliers. Only the variables ‘correct showing’ (*n* = 10) and ‘showing effort’ (*n* = 5) displayed outliers.

Table E1

Results of model comparisons for data sets adjusted for outliers

**Comparison**	**AIC**	**χ^2**	***df***	***p***
Success ~ correct showing + session/trial/phase				
Null model	585.50			
+ session/trial/phase	**585.87**	**9.63**	**5**	**.087**
+ correct showing	*533.29*	*54.58*	*1*	*< .001*
Showing effort ~ session/trial/phase				
Null model	-265.77			
+ session/trial/phase	*-354.01*	*98.24*	*5*	*< .001*
+ condition	-354.32	2.31	1	.129
+ condition:session/trial/phase	-345.25	0.93	5	.968

Note. The null model only contained pair ID as random intercept. Significant results are printed in italics. Significances differing from the main analyses are printed in bold.

Table E2

Result summary of the final GLMM for the effect of correct showing on success adjusted for outliers

**Random effects**	**Variance**		***SD***	
ID (Intercept)	0.86		0.93	

**Fixed effects**	**β**	***SE***	***z***	***p***
(Intercept)	-0.78	0.51	-1.52	.128
correct showing	*7.28*	*1.09*	*6.66*	*< .001*
session	-0.45	0.56	-0.89	.419
session1:trial	*-0.36*	*0.15*	*-2.36*	*.019*
session2:trial	-0.14	0.15	-0.89	.372
session1:trial:phase	0.10	0.12	0.83	.406
session2:trial:phase	0.10	0.12	0.89	.376

Note. Wald's *z*-score = β / *SE*, significant results are printed in italics.

Table E3

Result summary of the final LMM for the effect of session/trial/phase on showing effort adjusted for outliers

**Random effects**	**Variance**	***SD***
ID (Intercept)	0.00	0.07

**Fixed effects**	**β**	***SE***	***t***	***df***	***p***
(Intercept)	*0.38*	*0.03*	*13.42*	*285.30*	*< .001*
session	0.02	0.04	0.58	412.89	.564
session1:trial	0.01	0.01	1.08	413.48	.280
session2:trial	-0.01	0.01	-0.59	412.47	.557
session1:trial:phase	*-0.05*	*0.01*	*-6.38*	*415.62*	*< .001*
session2:trial:phase	*-0.06*	*0.01*	*-7.29*	*416.42*	*< .001*

Note. Significant results are printed in italics.

Appendix F

Result summary of all LMMs calculated for model comparisons for analyses regarding the effects of owner behaviour on correct showing and showing effort

Table F1

Result summary of the LMMs calculated for model comparisons regarding the effect owners' behaviour as well as condition, session, trial and phase on the proportion of correct showing

**Fixed effects**	**β**	***SE***	***t***	***df***	***p***
Null model					
(Intercept)	*0.26*	*0.01*	*25.65*	*28.35*	*< .001*
+ owner behaviour					
(Intercept)	*0.27*	*0.02*	*16.53*	*110.60*	*< .001*
owner behaviour	*0.00*	*0.00*	*-2.78*	*250.90*	*.006*
+ condition					
(Intercept)	*0.27*	*0.02*	*16.53*	*110.60*	*< .001*
owner behaviour	*0.00*	*0.00*	*-2.58*	*252.20*	*.011*
condition	*0.03*	*0.01*	*2.74*	*424.10*	*.006*
+ owner behaviour:condition (final m.)					
(Intercept)	*0.25*	*0.02*	*13.31*	*177.50*	*< .001*
owner behaviour	0.00	0.00	-0.39	388.00	.694
condition	*0.08*	*0.02*	*3.80*	*432.10*	*< .001*
owner behaviour:condition	*0.00*	*0.00*	*-2.75*	*436.70*	*.006*
+ session/trial/phase					
(Intercept)	*0.29*	*0.03*	*10.80*	*369.10*	*< .001*
owner behaviour	0.00	0.00	-0.72	362.40	.469
condition	*0.08*	*0.02*	*3.77*	*427.30*	*< .001*
owner behaviour:condition	*0.00*	*0.00*	*-2.72*	*432.00*	*.007*
S	-0.03	0.03	-1.14	418.00	.255
S1:T	-0.01	0.01	-1.24	420.40	.217
S2:T	0.00	0.01	-0.27	418.60	.791
S1:T:P	-0.01	0.01	-1.28	429.60	.201
S2:T:P	-0.01	0.01	-1.08	435.00	.280

(continued)

Table F1

Result summary of the LMMs calculated for model comparisons regarding the effect owners' owner behaviour as well as condition, session, trial and phase on the proportion of correct showing (continued)

**Fixed effects**	**β**	***SE***	***t***	***df***	***p***
+ owner behaviour:session/trial/phase					
(Intercept)	*0.26*	*0.02*	*11.95*	*255.50*	*< .001*
owner behaviour	0.00	0.00	0.11	443.80	.909
condition	*0.08*	*0.02*	*3.69*	*426.10*	*< .001*
owner behaviour:condition	*0.00*	*0.00*	*-2.62*	*430.80*	*.009*
S	-0.02	0.02	-0.96	427.80	.335
owner behaviour:S	0.00	0.00	-0.25	424.90	.804
owner behaviour:S1:T	0.00	0.00	-0.31	420.80	.756
owner behaviour:S2:T	0.00	0.00	0.41	418.40	.685
owner behaviour:S1:T:P	0.00	0.00	-1.68	428.30	.093
owner behaviour:S2:T:P	0.00	0.00	-0.81	432.40	.421

Note. In all models the random intercept pair ID had a variance of 0.00 and a standard deviation od 0.04. S = session, T = trial, P = phase, significant coefficients are printed in italics.

Table F2

Result summary of the LMMs calculated for model comparisons regarding the effect owners' behaviour as well as condition, session, trial and phase on showing effort

**Fixed effects**	**β**	***SE***	***t***	***df***	***p***
+ owner behaviour					
(Intercept)	*0.36*	*0.03*	*11.00*	*285.40*	*< .001*
owner behaviour	0.00	0.00	1.83	274.70	.069
S	0.13	0.07	1.79	416.90	.074
S1:T	0.01	0.01	0.87	418.90	.382
S2:T	-0.02	0.01	-1.83	416.40	.068
S1:T:P	*-0.05*	*0.01*	*-6.12*	*427.90*	*< .001*
S2:T:P	*-0.02*	*0.00*	*-6.89*	*435.10*	*< .001*
+ condition					
(Intercept)	*0.35*	*0.03*	*10.24*	*300.70*	*< .001*
owner behaviour	0.00	0.00	1.92	273.60	.055
condition	0.02	0.02	1.27	416.20	.205
S	0.13	0.07	1.80	415.90	.073
S1:T	0.01	0.01	0.88	417.90	.380
S2:T	-0.02	0.01	-1.84	415.40	.067
S1:T:P	*-0.05*	*0.01*	*-6.12*	*426.90*	*< .001*
S2:T:P	*-0.02*	*0.00*	*-6.88*	*434.10*	*< .001*
+ owner behaviour:condition (final m.)					
(Intercept)	*0.38*	*0.04*	*10.58*	*337.50*	*< .001*
owner behaviour	0.00	0.00	0.19	384.60	.849
condition	-0.04	0.03	-1.45	421.40	.149
owner behaviour:condition	*0.00*	*0.00*	*2.52*	*425.90*	*.012*
S	0.13	0.07	1.80	414.90	.072
S1:T	0.01	0.01	0.90	416.90	.367
S2:T	-0.02	0.01	-1.92	414.40	.056
S1:T:P	*-0.05*	*0.01*	*-6.23*	*425.70*	*< .001*
S2:T:P	*-0.02*	*0.00*	*-6.65*	*433.80*	*< .001*

(continued)

Table F2

Result summary of the LMMs calculated for model comparisons regarding the effect owners' behaviour as well as condition, session, trial and phase on showing effort (continued)

**Fixed effects**	**β**	***SE***	***t***	***df***	***p***
+ owner behaviour:session/trial/phase					
(Intercept)	*0.33*	*0.03*	*11.21*	*229.00*	*< .001*
owner behaviour	0.00	0.00	1.33	440.90	.183
condition	-0.06	0.03	-1.94	420.10	.053
owner behaviour:condition	*0.00*	*0.00*	*3.10*	*424.70*	*.002*
S	-0.04	0.03	-1.39	421.80	.166
owner behaviour:S	0.00	0.00	1.67	416.20	.096
owner behaviour:S1:T	0.00	0.00	0.79	416.40	.431
owner behaviour:S2:T	0.00	0.00	-1.31	414.30	.191
owner behaviour:S1:T:P	*0.00*	*0.00*	*-5.70*	*423.30*	*< .001*
owner behaviour:S2:T:P	*0.00*	*0.00*	*-5.87*	*429.50*	*< .001*

Note. In all models the random intercept pair ID had a variance of 0.00 and a standard deviation od 0.07. S = session, T = trial, P = phase, significant coefficients are printed in italics.

Appendix G

Results of sensitivity analyses for analyses regarding the effects of owner behaviour on correct showing and showing effort

Table G1

Results of model comparisons for the effect of owner behaviour on correct showing for data sets excluding problem trials, problem pairs and rows with retrieval, respectively

**Comparison**	**AIC**	χ^2	***df***	***p***
Problem trials excluded (*n* = 10)[a]				
Null model	-500.93			
+ owner behaviour	*-507.74*	*8.81*	*1*	*.003*
+ condition	*-511.71*	*5.97*	*1*	*.015*
+ owner behaviour:condition (final m.)	*-515.99*	*6.28*	*1*	*.012*
+ session/trial/phase	-512.50	6.51	5	.260
+ owner behaviour:session/trial/phase	-509.16	0.00	1	1.00
Problem pairs excluded (*n* =7)[b]				
Null model	-355.93			
+ owner behaviour	*-360.91*	*6.97*	*1*	*.008*
+ condition	**-362.21**	**3.31**	**1**	**.069**
+ owner behaviour:condition (final m.)	**-363.60**	**3.38**	**1**	**.066**
+ session/trial/phase	-362.08	8.49	5	.131
+ owner behaviour:session/trial/phase	-358.18	0.00	1	1.00
Retrieval excluded (*n* =26)				
Null model	-495.96			
+ owner behaviour	*-501.06*	*7.10*	*1*	*.008*
+ condition	*-508.71*	*9.65*	*1*	*.002*
+ owner behaviour:condition (final m.)	*-517.76*	*11.06*	*1*	*.001*
+ session/trial/phase	-515.97	8.21	5	.145
+ owner behaviour:session/trial/phase	-514.14	0.17	1	.677

Note. The null model only contained pair ID as random intercept. Significant results are printed in italics. Significances differing from the main explorative analyses are printed in bold.

[a] corresponds to 20 data rows

[b] corresponds to 112 data rows

Table G2

Result summary of the final LMM for the effect of owner behaviour on correct showing for data sets excluding problem trials, problem pairs and rows with retrieval, respectively

**Fixed effects**	**β**	***SE***	***t***	***df***	***p***
Problem trials excluded (*n* = 10)[a]					
(Intercept)	*0.25*	*0.02*	*13.45*	*182.70*	*< .001*
owner behaviour	0.00	0.00	-0.72	366.60	.475
condition	*0.08*	*0.02*	*3.43*	*414.50*	*.001*
owner behaviour:condition	*0.00*	*0.00*	*-2.50*	*418.20*	*.013*
Problem pairs excluded (*n* = 7)[b]					
(Intercept)	*0.27*	*0.02*	*11.93*	*135.40*	*< .001*
owner behaviour	0.00	0.00	-0.90	292.80	.370
condition	*0.07*	*0.03*	*2.53*	*325.00*	*.012*
owner behaviour:condition	**0.00**	**0.00**	**-1.83**	**328.40**	**.068**
Retrieval excluded (*n* =26)					
(Intercept)	*0.23*	*0.02*	*11.83*	*196.90*	*< .001*
owner behaviour	0.00	0.00	0.18	382.20	.861
condition	*0.11*	*0.02*	*4.48*	*411.10*	*< .001*
owner behaviour:condition	*0.00*	*0.00*	*-3.33*	*413.40*	*.001*

Note. In all models the random intercept was pair ID with a variance of 0.00 and a standard deviation of 0.04. Significant results are printed in italics. Significances differing from the main explorative analyses are printed in bold.

[a] corresponds to 20 data rows

[b] corresponds to 112 data rows

Table G3

Results of model comparisons for the effect of owner behaviour on showing effort for data sets excluding problem trials, problem pairs and rows with retrieval, respectively

**Comparison**	**AIC**	χ^2	***df***	***p***
Problem trials excluded (*n* = 10)[a]				
ID + session/trial/phase[b]	-288.86			
+ owner behaviour	***-291.40***	***4.54***	***1***	***.033***
+ condition	-290.62	1.22	1	.270
+ owner behaviour:condition	*-296.25*	*7.64*	*1*	*.006*
+ owner behaviour:session/trial/phase	-282.10	0.00	1	1.00
Problem pairs excluded (*n* =7)[c]				
ID + session/trial/phase[b]	-206.59			
+ owner behaviour	-205.99	1.40	1	.237
+ condition	-204.11	0.12	1	.725
+ owner behaviour:condition	*-211.05*	*8.94*	*1*	*.003*
+ owner behaviour:session/trial/phase	-201.27	0.00	1	1.00
Retrieval excluded (*n* =26)				
ID + session/trial/phase[b]	-299.44			
+ owner behaviour	-299.90	2.46	1	.117
+ condition	-300.47	2.57	1	.109
+ owner behaviour:condition	*-302.88*	*4.42*	*1*	*.036*
+ owner behaviour:session/trial/phase	-291.48	0.00	1	1.00

Note. Significant results are printed in italics. Significances differing from the main analysis are printed in bold.

[a] corresponds to 20 data rows

[b] Model comparisons were based on the final model investigating the effects of condition, session, trial and phase on showing since this analysis already showed that session/trial/phase significantly predicted showing effort.

[c] corresponds to 111 data rows

Table G4

Result summary of the final LMM for the effect of owner behaviours on showing effort for data sets excluding problem trials, problem pairs and rows with retrieval, respectively

**Fixed effects**	**β**	***SE***	***t***	***df***	***p***
Problem trials excluded (*n* = 10)[a]					
(Intercept)	*0.38*	*0.04*	*10.33*	*336.30*	*< .001*
owner behaviour	0.00	0.00	0.29	362.00	.769
condition	-0.05	0.03	-1.74	402.80	.083
owner behaviour:condition	*0.00*	*0.00*	*2.75*	*406.80*	*.006*
session	0.10	0.07	1.42	395.00	.156
session1:trial	0.01	0.01	0.86	397.70	.388
session2:trial	-0.02	0.01	-1.57	394.40	.117
session1:trial:phase	*-0.05*	*0.01*	*-6.41*	*404.50*	*< .001*
session2:trial:phase	*-0.02*	*0.00*	*-6.18*	*413.50*	*< .001*
Problem pairs excluded (*n* = 7)[b]					
(Intercept)	*0.40*	*0.04*	*9.70*	*273.70*	*< .001*
owner behaviour	0.00	0.00	-0.76	263.00	.448
condition	***-0.08***	***0.03***	***-2.30***	***317.60***	***.022***
owner behaviour:condition	*0.00*	*0.00*	*2.97*	*321.50*	*.003*
session	0.13	0.08	1.58	311.30	.116
session1:trial	0.02	0.01	1.35	313.20	.178
session2:trial	-0.02	0.01	-1.60	310.60	.110
session1:trial:phase	*-0.06*	*0.01*	*-6.45*	*321.30*	*< .001*
session2:trial:phase	*-0.02*	*0.00*	*-4.98*	*328.70*	*< .001*
Retrieval excluded (*n* =26)					
(Intercept)	*0.36*	*0.04*	*9.68*	*335.00*	*< .001*
owner behaviour	0.00	0.00	0.26	386.00	.797
condition	-0.03	0.03	-0.96	399.60	.340
owner behaviour:condition	*0.00*	*0.00*	*2.08*	*402.20*	*.038*
session	0.11	0.07	1.60	391.00	.110
session1:trial	0.02	0.01	1.58	394.00	.114
session2:trial	-0.01	0.01	-1.34	390.20	.181
session1:trial:phase	*-0.05*	*0.01*	*-6.32*	*398.20*	*< .001*
session2:trial:phase	*-0.02*	*0.00*	*-6.74*	*404.50*	*< .001*

Note. In all models the random intercept was pair ID with a variance of 0.00 and a standard deviation of 0.06. Significant results are printed in italics. Significances differing from the main explorative analyses are printed in bold.

[a] corresponds to 20 data rows

[b] corresponds to 111 data rows

Appendix H

Results of analyses regarding the effects of owner behaviour on correct showing and showing effort adjusted for outliers.

Table H1

Results of model comparisons for the effect of owner behaviour on correct showing adjusted for outliers regarding correct showing and owner behaviour, respectively

**Comparison**	**AIC**	χ^2	***df***	***p***
Outliers correct showing excluded (*n* = 10)				
Null model	-604.72			
+ owner behaviour	**-606.57**	**3.85**	**1**	**.050**
+ condition	**-607.76**	**3.19**	**1**	**.074**
+ owner behaviour:condition (final m.)	*-611.24*	*5.48*	*1*	*.019*
+ session/trial/phase	-609.63	8.39	5	.136
+ owner behaviour:session/trial/phase	-604.78	0.00	1	1.00
Outliers owner behaviour excluded (*n* = 2)				
Null model	-525.83			
+ owner behaviour	*-531.62*	*7.79*	*1*	*.005*
+ condition	*-536.90*	*7.28*	*1*	*.007*
+ owner behaviour:condition (final m.)	*-543.21*	*8.31*	*1*	*.004*
+ session/trial/phase	-541.52	8.31	5	.140
+ owner behaviour:session/trial/phase	-539.92	0.40	1	.526

Note. The null model only contained pair ID as random intercept. Sample sizes indicate the respective number of excluded data rows. Significant results are printed in italics. Significances differing from the main explorative analyses are printed in bold.

Table H2

Result summary of final the LMM for the effect of owner behaviour on correct showing adjusted for outliers regarding correct showing and owner behaviour, respectively

**Random intercepts**[a]		**Variance**		**SD**	
Outliers correct showing excluded (*n* = 10)		0.00		0.04	
Outliers owner behaviour excluded (*n* = 2)		0.00		0.03	
**Fixed effects**	**β**	***SE***	***t***	***df***	***p***
Outliers correct showing excluded (*n* = 10)					
(Intercept)	*0.25*	*0.02*	*13.17*	*179.80*	*< .001*
owner behaviour	0.00	0.00	-0.34	386.80	.738
condition	*0.09*	*0.02*	*3.89*	*430.80*	*< .001*
owner behaviour:condition	*0.00*	*0.00*	*-2.88*	*435.50*	*.004*
Outliers owner behaviour excluded (*n* = 2)					
(Intercept)	*0.24*	*0.02*	*14.28*	*181.10*	*< .001*
owner behaviour	0.00	0.00	-0.05	366.30	.961
condition	*0.06*	*0.01*	*2.93*	*425.70*	*.004*
owner behaviour:condition	*0.00*	*0.00*	*-2.33*	*429.80*	*.020*

Note. The variable 'talking' did not display outliers. Sample sizes indicate the respective number of excluded data rows. Significant results are printed in italics. Significances differing from the main analyses are printed in bold.

[a] In all models the random intercept was pair ID.

Table H3

Results of model comparisons for the effect of owner behaviour on showing effort adjusted for outliers regarding showing effort and owner behaviour, respectively

**Comparison**	**AIC**	χ^2	***df***	***p***
Outliers showing effort excluded (*n* = 5)				
ID + session/trial/phase[a]	-369.40			
+ owner behaviour	***-372.90***	***5.49***	***1***	***.019***
+ condition	-374.00	3.10	1	.078
+ owner behaviour:condition	*-377.80*	*5.81*	*1*	*.016*
+ owner behaviour:session/trial/phase	-363.89	0.00	1	1.00
Outliers owner behaviour excluded (*n* = 2)				
ID + session/trial/phase[a]	-299.82			
+ owner behaviour	-301.22	3.40	1	.065
+ condition	-300.95	1.73	1	.189
+ owner behaviour:condition	*-306.46*	*7.51*	*1*	*.006*
+ owner behaviour:session/trial/phase	-290.59	0.00	1	1.00

Note. The null model only contained pair ID as random intercept. Sample sizes indicate the respective number of excluded data rows. Significant results are printed in italics. Significances differing from the main analyses are printed in bold.

[a] Model comparisons were based on the final model investigating the effects of condition, session, trial and phase on showing since this analysis already showed that session/trial/phase significantly predicted showing effort.

Table H4

Result summary of final the LMM for the effect of owner behaviour on showing effort adjusted for outliers regarding showing effort and owner behaviour, respectively

**Fixed effects**	**β**	***SE***	***t***	***df***	***p***
Outliers showing effort excluded (*n* = 5)					
(Intercept)	*0.36*	*0.03*	*10.55*	*325.80*	*< .001*
owner behaviour	0.00	0.00	0.74	392.30	.460
condition	-0.03	0.03	-1.08	416.30	.281
owner behaviour:condition	*0.00*	*0.00*	*2.39*	*420.20*	*.017*
session	***0.14***	***0.06***	***2.24***	***410.20***	***.025***
session1:trial	0.01	0.01	0.89	413.10	.375
session2:trial	***-0.02***	***0.01***	***-2.28***	***409.60***	***.023***
session1:trial:phase	*-0.05*	*0.01*	*-6.11*	*420.60*	*< .001*
session2:trial:phase	*-0.02*	*0.00*	*-7.30*	*428.00*	*< .001*
Outliers owner behaviour excluded (*n* = 2)					
(Intercept)	*0.38*	*0.04*	*10.47*	*335.00*	*< .001*
owner behaviour	0.00	0.00	0.12	383.00	.903
condition	-0.05	0.03	-1.62	420.00	.106
owner behaviour:condition	*0.00*	*0.00*	*2.72*	*424.80*	*.007*
session	0.13	0.07	1.83	412.70	.068
session1:trial	0.01	0.01	0.94	414.60	.346
session2:trial	-0.02	0.01	-1.95	412.30	.052
session1:trial:phase	*-0.05*	*0.01*	*-6.05*	*425.10*	*< .001*
session2:trial:phase	*-0.02*	*0.00*	*-6.52*	*432.30*	*< .001*

Note. In all models the random intercept pair ID had a variance of 0.00 and a standard deviation of 0.07. Sample sizes indicate the respective number of excluded data rows. Significant results are printed in italics. Significances differing from the main analyses are printed in bold.

Appendix I

Results of model comparisons

Table I1

Results of model comparisons for the effect of correct showing on success

**Comparison**	**AIC**	χ^2	***df***	***p***
Null model	594.99			
+ session/trial/phase	593.62	11.37	5	.045 *
+ correct showing	540.42	55.20	1	<.001 ***

Note. The null model only contained pair ID as random intercept. Significance code: p < .05 *, < .001 ***

Table I2

Results of model comparisons for the effect of condition, session, trial and phase on success

**Comparison**	**AIC**	χ^2	***df***	***p***
Null model	613.87			
+ session/trial/phase	611.33	12.54	5	.028 *
+ condition	599.33	14.00	1	< .001 ***
+ condition:session/trial/phase	606.39	2.94	1	.709

Note. The null model only contained pair ID as random intercept. Significance code: p < .05 *, < .001 ***

Table I3

Results of model comparisons for the effect of condition, session, trial and phase on showing effort

**Comparison**	**AIC**	χ^2	***df***	***p***
Null model	-210.59			
+ session/trial/phase	-290.12	189.53	5	< .001 ***
+ condition	-289.35	1.23	1	.267
+ condition:session/trial/phase	-280.04	0.69	5	.984

Note. The null model only contained pair ID as random intercept. Significance code: p < .001 ***

Table I4

Results of model comparisons for the effect of owner behaviour on correct showing

**Comparison**	**AIC**	χ^2	***df***	***p***
Null model	-530.03			
+ owner behaviour	-535.76	7.73	1	.005 **
+ condition	-541.22	7.46	1	.006 **
+ owner behaviour:condition	-546.77	7.55	1	.006 **
+ session/trial/phase	-544.38	7.61	5	.179
+ owner behaviour:session/trial/phase	-40.63	0.00	1	1.00

Note. The null model only contained pair ID as random intercept. Significance code: $p < .01$ **

Table I5

Results of model comparisons for the effect of owner behaviours on showing effort

**Comparison**	**AIC**	χ^2	***df***	***p***
ID + session/trial/phase[a]	-303.12			
+ owner behaviour	-304.50	3.38	1	.066
+ condition	-304.14	1.63	1	.201
+ owner behaviour:condition	-308.55	6.42	1	.011 *
+ owner behaviour:session/trial/phase	-294.05	0.00	1	1.00

Note. Significance code: $p < .05$ *

[a] Model comparisons were based on the final model investigating the effects of condition, session, trial and phase on showing since this analysis already showed that session/trial/phase significantly predicted showing effort.

Table I6

Results of model comparisons for the effect of separated owner behaviours on correct showing

**Comparison**	**AIC**	χ^2	***df***	***p***
Null model	-530.03			
+ prompting	-542.22	14.20	1	< .001 ***
+ talking	-542.14	1.91	1	.167
+ calling	-540.81	0.68	1	.411
+ prompting:talking[a]	-541.65	2.84	1	.092
+ prompting:talking:calling[a]	-539.33	3.68	1	.298
+ condition	-545.61	6.79	1	.009 **
+ prompting:condition	-547.58	3.97	1	.046 *
+ talking:condition	-552.81	7.24	1	.007 **
+ calling:condition[a]	-550.83	0.02	1	.893
+ session/trial/phase	-548.93	6.12	5	.295
+ prompting:session/trial/phase	-543.48	0.00	1	1.00
+ talking:session/trial/phase	-534.19	0.71	5	.983
+ calling:session/trial/phase	-527.11	2.92	5	.712
- calling[b]	-552.81	0.96	1	.327

Note. The null model only contained pair ID as random intercept. Significance code: p < .05 *, < .01 **, < .001 ***

[a] These factors were later omitted again, i.e. subsequent factors were added to the respective previous model without this specific note

[b] This model was based on the final model, i.e. it included prompting, talking, condition, prompting*condition and talking*condition.

Table I7

Results of model comparisons for the effect of separated owner behaviours on showing effort

**Comparison**	**AIC**	χ^2	***df***	***p***
ID + session/trial/phase[a]	-303.12			
+ prompting	-302.60	1.48	1	.224
+ talking	-303.33	2.73	1	.099
+ calling	-302.57	1.24	1	.266
+ condition	-302.18	1.61	1	.204
+ prompting:condition	-312.36	12.18	1	< .001 ***
+ talking:condition	-311.56	1.20	1	.273
+ calling:condition	-310.81	1.25	1	.264
+ prompting:condition *S/T/P	-294.44	0.00	7	1.00
+ talking:condition *S/T/P	-282.33	7.90	10	.639
+ calling:condition *S/T/P	-276.58	14.25	10	.162

Note. Significance code: p < .001 ***, S = session, T = trial, P = phase.

[a] Model comparisons were based on the final model investigating the effects of condition, session, trial and phase on showing since this analysis already showed that session/trial/phase significantly predicted showing effort.

Appendix J

Results of analyses regarding the effects of owner behavior for the separated variables non-verbal prompting, talking and calling the dog by its name

Table J1

Result summary of the final LMM for the effect of the owner's non-verbal prompting, talking and calling the dog's name and condition on the proportion of correct showing

**Random effects**	**Variance**	***SD***
ID (Intercept)	0.00	0.04

**Fixed effects**	**β**	***SE***	***t***	***df***	***p***
(Intercept)	0.25	0.02	13.12	167.90	< .001 ***
prompting	0.00	0.00	-2.93	419.70	.004 **
talking	0.00	0.00	2.97	406.10	.003 **
calling	0.00	0.00	-0.98	374.50	.326
condition	0.09	0.02	3.90	428.10	< .001 ***
prompting:condition	0.00	0.00	-0.11	434.90	.909
talking:condition	0.00	0.00	-2.68	425.40	.008 **

Note. Significance code: $p < .05$ *, < .01 **, < .001 ***

Table J2

Result summary of the final LMM for the effect of the owner's non-verbal prompting, talking, condition, session, trial and phase on showing effort

**Random effects**	**Variance**	***SD***
ID (Intercept)	0.00	0.07

**Fixed effects**	**β**	***SE***	***t***	***df***	***p***
(Intercept)	0.39	0.04	10.91	316.30	< .001 ***
prompting	0.00	0.00	-1.83	421.80	.068
talking	0.00	0.00	1.20	301.40	.231
calling	0.01	0.00	1.36	405.00	.176
condition	-0.06	0.03	-2.12	420.00	.035 *
prompting:condition	0.00	0.00	3.47	426.00	.001 **
session	0.13	0.07	1.92	412.60	.056
session1:trial	0.01	0.01	0.58	422.50	.563
session2:trial	-0.02	0.01	-2.14	414.40	.033 *
session1:trial:phase	-0.05	0.01	-5.08	437.60	< .001 ***
session2:trial:phase	-0.02	0.00	-5.42	439.90	< .001 ***

Note. Significance code: $p < .05$ *, < .01 **, < .001 ***
